# Biosynthesis and Regulatory Mechanisms of Plant Flavonoids: A Review

**DOI:** 10.3390/plants14121847

**Published:** 2025-06-16

**Authors:** Yuye Mao, Jiajia Luo, Zeping Cai

**Affiliations:** 1 School of Tropical Agriculture and Forestry, Hainan University, Haikou 570228, China; 20223006839@hainanu.edu.cn (Y.M.); luojiajia@catas.cn (J.L.); 2 Tropical Crops Genetic Resources Institute, Chinese Academy of Tropical Agricultural Sciences, Haikou 571101, China

**Keywords:** flavonoids, biosynthesis, regulation, transcription factors, gene expression

## Abstract

Flavonoids are a class of secondary metabolites synthesized by plants, characterized by a C6-C3-C6 carbon skeleton and derived from the phenylpropane metabolism pathway. They play crucial biological roles, not only in plant pigment production and responses to biotic and abiotic stresses but also in medicinal applications. Consequently, the biosynthesis and regulatory mechanisms of flavonoids have been a focal point in plant transcription and gene expression research. The biosynthetic pathways of flavonoids include branches such as isoflavones, flavones, flavonols, anthocyanins, and proanthocyanidins, with some pathways and key enzymes already well-characterized. Studies indicate that plant flavonoids are regulated by various factors, including transcription factors, non-coding endogenous small RNAs (miRNAs), and plant hormones. This review systematically summarizes the structure and classification of plant flavonoids, their biosynthetic and regulatory mechanisms, and the factors influencing flavonoid synthesis. By discussing the regulation of flavonoid-related gene expression in plants, this work provides valuable insights and a theoretical foundation for future research and applications of flavonoids.

## 1. Introduction

Flavonoids, an important class of plant secondary metabolites, are ubiquitously distributed in vascular plants. They play pivotal roles in plant pigment production, responses to biotic and abiotic stresses, and exhibit significant medicinal value. The evolutionary origin of flavonoids can be traced back to pioneering land plants [1], with subsequent refinement and differentiation during plant evolution. Early studies suggested that mosses and liverworts were the most ancient plants producing flavonoid compounds, while algae were generally considered devoid of flavonoids. However, recent research has identified flavonoids in a few evolutionary divergent lineages of microalgae, including *Cyanobacteria*, *Rhodophyta*, *Chlorophyta*, *Haptophyta*, and *Ochrophyta* [2]. As plants gradually transitioned from aquatic to terrestrial environments, their flavonoid compounds underwent extensive modifications. From an evolutionary perspective, the emergence of flavonoids represents an adaptive response to the more diverse, irregular, and multifactorial stress conditions on land. When subjected to stresses such as UV-B radiation, drought, cold, or insect herbivory, the hydroxyl groups in flavonoid structures confer strong free radical-scavenging capacity, reducing reactive oxygen species (ROS) accumulation. This enhances the plant’s antioxidant capacity and stress resistance, mitigating oxidative damage. These adaptations were crucial prerequisites for plants to colonize terrestrial habitats [3]. As terrestrial environments changed and plants evolved into higher plants, the biosynthetic pathways and regulatory mechanisms of flavonoids became increasingly sophisticated. These compounds now participate in diverse physiological processes including plant growth, development, and stress resistance, enabling adaptation to varying ecological niches. For example, with the evolution from gymnosperms to angiosperms, flavonoid compounds such as anthocyanins gradually became more abundant in flowering plants. The accumulation of these compounds provides angiosperms with the appropriate colors needed to attract pollinators. Under typical cellular conditions, the most common types of anthocyanins found in the flowers and fruits of seed plants do not typically produce a ‘sky-blue’. However, certain pollinators or seed dispersal agents, particularly some bee species, exhibit a preference for sky-blue. Consequently, to adapt to these preferences, plants have evolved diverse anthocyanin structures and cellular mechanisms [4]. Additionally, color production can deter attacks by certain insect herbivores [3]. Flavonoid compounds can regulate polar auxin transport (PAT) by acting on the auxin efflux carrier PIN-FORMED (PIN) through ATP-binding cassette subfamily B/P-glycoprotein (ABCB/PGP) transporters, enabling plants to respond to stress in a more advantageous manner [3]. Studies have found that AtABCB/PGP4 catalyzes auxin transport in roots. As a target of flavonoid regulation, AtABCB4 is often partially inhibited, affecting auxin migration in the elongation zone and thereby modulating root growth and development. The absence of flavonoid compounds leads to reduced concentrations of inhibitory auxin in this region, while increased flavonoid levels accelerate auxin accumulation [3]. Therefore, research on flavonoids provides crucial insights into the biosynthetic and regulatory mechanisms of plant secondary metabolites and their functional roles in plant adaptive evolution.

Furthermore, flavonoids serve as crucial bioactive substances that mediate diverse physiological regulatory functions in animals. Substantial evidence indicates that flavonoids effectively inhibit the initial inflammatory cascade [5], demonstrating significant anti-inflammatory properties. Additionally, their potent free radical scavenging capacity provides robust cellular protection against oxidative damage [6]. Studies have found that flavonoid compounds also exhibit significant antibacterial effects and can combat bacterial infections through multiple mechanisms, including inhibition of bacterial cell wall synthesis, disruption of bacterial cell membranes, induction of oxidative stress, suppression of protein synthesis, and interference with bacterial DNA replication and transcription [7]. Given animals’ inherent inability to biosynthesize flavonoids, comprehensive elucidation of plant flavonoid metabolic pathways and their regulatory networks establishes a critical foundation for biotechnological approaches to enhance flavonoid content in agricultural crops and human dietary sources. This review systematically summarizes current knowledge on flavonoid biosynthesis and its regulatory mechanisms, while providing novel insights for future research on plant transcription and gene expression.

## 2. Structural Characteristics and Classification of Flavonoids

**Figure 1:**
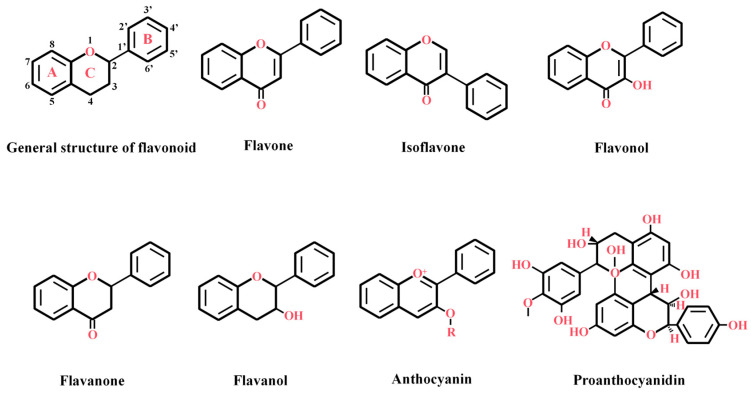
Structure and classification of flavonoids. A and B are 6-carbon benzene rings, and C is 3-carbon heterocyclic ring.

Flavonoids represent a major class of plant secondary metabolites synthesized through the phenylpropanoid metabolism pathway. To date, more than 9000 plant-derived flavonoid compounds have been isolated and structurally characterized [8]. Flavonoids are characterized by a basic structure consisting of a C6-C3-C6 carbon skeleton (Figure 1), which comprises two 6-carbon benzene rings (Ring A and Ring B) linked by a 3-carbon heterocyclic ring (Ring C) [9]. Based on the oxidation state of the 3-carbon heterocyclic ring and the number of substituents attached to the benzene ring, flavonoids can be broadly classified into seven subgroups: flavones, isoflavones, flavonols, flavanones, flavanols, anthocyanins, and proanthocyanidins (Figure 1). Flavonoids play crucial roles in plant growth and development. For example, flavones contribute to drought resistance and protect plants from UV-B radiation [10]. Flavonols have been demonstrated to be essential for pollen germination and pollen tube formation [11] and regulate the polar transport of auxin by mediating the stabilization of PIN efflux complexes [12]. Anthocyanins, a class of naturally occurring water-soluble pigments, are widely distributed in the vacuoles of flowers, fruits, stems, and leaves, exhibiting diverse biological activities such as free radical scavenging [13].

## 3. Biosynthesis of Plant Flavonoids

### 3.1. Flavonoid Biosynthetic Pathway

**Figure 2:**
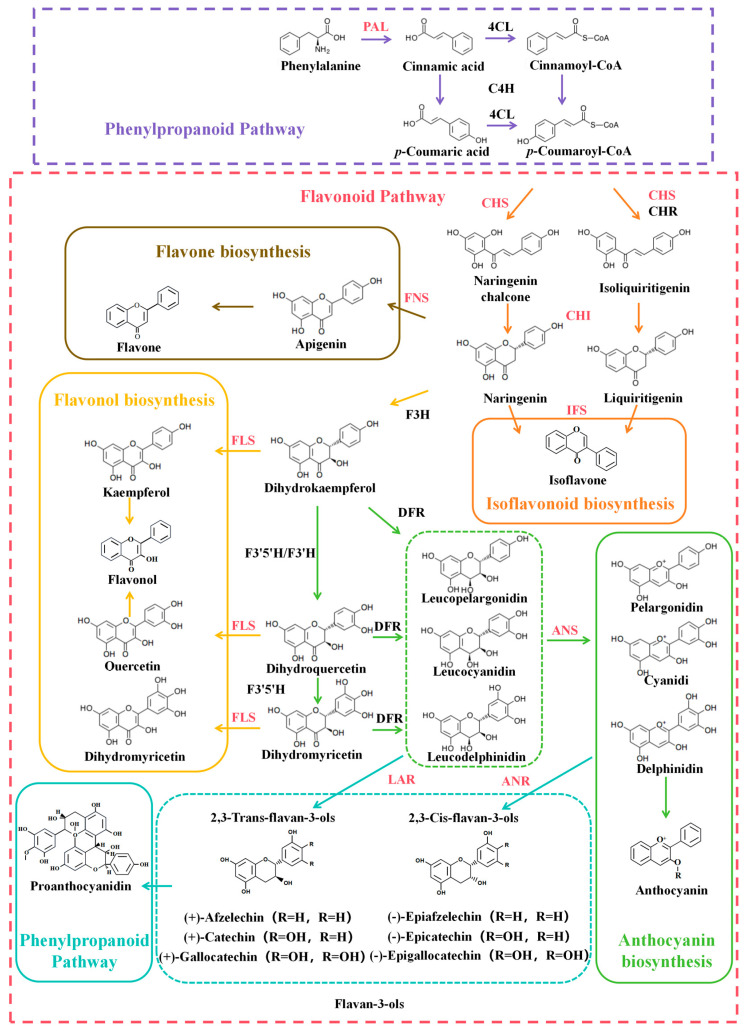
The biosynthesis pathways of flavonoids in plants. Flavonoid biosynthesis consists of two parts: the early phenylpropanoid pathway and the flavonoid biosynthesis pathway. The enzymes highlighted in red in the figure are rate-limiting enzymes. The enzyme abbreviations are as follows: PAL, phenylalanine ammonia-lyase; C4H, cinnamic acid 4-hydroxylase; 4CL, 4-coumarate-CoA ligase; CHS, chalcone synthase; CHR, chalcone reductase; CHI, chalcone isomerase; IFS, isoflavonoid synthase; FNS, flavone synthase; F3H, flavanone 3-hydroxylase; FLS, flavonol synthase; F3′5′H, flavonoid 3′,5′-hydroxylase; F3′H, flavonoid 3′-hydroxylase; DFR, dihydroflavonol 4-reductase; ANS, anthocyanidin synthase; LAR, leucoanthocyanidin reductase; ANR, anthocyanidin reductase.

The flavonoid biosynthetic pathway comprises multiple branches, including isoflavone biosynthesis, flavone and flavonol biosynthesis, anthocyanin biosynthesis, and proanthocyanidin biosynthesis (Figure 2). The resulting flavonoid products undergo further modifications such as glycosylation, acylation, and methylation [14], ultimately generating diverse plant secondary metabolites.

Phenylpropanoid biosynthesis serves as the initial stage of flavonoid biosynthesis. In this process, phenylalanine is deaminated by phenylalanine ammonia-lyase (PAL) to yield cinnamic acid, which is subsequently converted to cinnamoyl-CoA via the action of 4-coumarate-CoA ligase (4CL). Meanwhile, both cinnamic acid and cinnamoyl-CoA can be hydroxylated by cinnamic acid 4-hydroxylase (C4H) to produce *p*-coumaric acid and *p*-coumaroyl-CoA, respectively. Additionally, *p*-coumaric acid can also be transformed into *p*-coumaroyl-CoA through 4CL activity. As the essential substrate, *p*-coumaroyl-CoA is channeled into the flavonoid biosynthetic pathway, initiating the formation of diverse flavonoid compounds.

*p*-Coumaroyl-CoA enters the flavonoid biosynthetic pathway where it is converted to naringenin chalcone by chalcone synthase (CHS), or to isoliquiritigenin through the combined action of CHS and chalcone reductase (CHR). Naringenin chalcone and isoliquiritigenin are subsequently isomerized by chalcone isomerase (CHI) to form naringenin and liquiritigenin, respectively. Naringenin and liquiritigenin serve as precursors entering the isoflavone biosynthetic pathway, where they are subsequently converted into various isoflavonoid compounds through the catalytic action of isoflavone synthase (IFS).

Naringenin serves as a key precursor for the biosynthesis of diverse flavonoids. Through the action of flavone synthase (FNS), it is converted to apigenin, which enters the flavone biosynthetic pathway. Subsequent modifications such as glycosylation then yield various derived flavone compounds.

Simultaneously, naringenin can be converted to dihydrokaempferol via flavanone 3-hydroxylase (F3H) catalysis. Dihydrokaempferol then serves as a substrate for either flavonoid 3′-hydroxylase (F3′H) or flavonoid 3′,5′-hydroxylase (F3′5′H), yielding dihydroquercetin through hydroxylation. Further F3′5′H-mediated hydroxylation of dihydroquercetin produces dihydromyricetin. These compounds (dihydrokaempferol, dihydroquercetin, and dihydromyricetin) represent key dihydroflavonol intermediates that are subsequently converted by flavonol synthase (FLS) into the corresponding flavonols: kaempferol, quercetin, and myricetin, respectively. These flavonols then enter the flavonol biosynthetic pathway for further structural modifications.

Dihydroflavonols represent a critical branch point in the flavonoid biosynthetic pathway. Under the catalysis of dihydroflavonol 4-reductase (DFR), they are converted to leucoanthocyanidins (leucopelargonidin, leucocyanidin, and leucodelphinidin). Anthocyanidin synthase (ANS) then catalyzes the formation of corresponding anthocyanidins (pelargonidin, cyanidin, and delphinidin) from these leucoanthocyanidins. These anthocyanidins subsequently enter the anthocyanin biosynthetic pathway, where glycosylation and other modifications produce stable anthocyanin pigments.

Simultaneously, leucopelargonidin, leucocyanidin, and leucodelphinidin are respectively converted by leucoanthocyanidin reductase (LAR) into the 2,3-trans-flavan-3-ols ((+)-afzelechin, (+)-catechin, and (+)-gallocatechin). In parallel, pelargonidin, cyanidin, and delphinidin are reduced by anthocyanidin reductase (ANR) to form the 2,3-cis-flavan-3-ols ((−)-epiafzelechin, (−)-epicatechin, and (−)-epigallocatechin). Both 2,3-trans-flavan-3-ols and 2,3-cis-flavan-3-ols (collectively termed flavan-3-ols) serve as direct precursors for proanthocyanidin biosynthesis, entering the proanthocyanidin pathway to generate diverse proanthocyanidins.

### 3.2. Key Enzymes in Flavonoid Biosynthesis

The International Enzyme Commission (EC) classifies enzymes into six major categories based on their catalytic reaction types: oxidoreductases, transferases, hydrolases, lyases, isomerases, and ligases. The biosynthesis of plant flavonoids involves all these enzyme classes, including oxidoreductases (e.g., IFS, FNS, F3H, FLS, DFR, ANS, LAR, ANR) responsible for oxidoreductase reactions, the transferase CHS catalyzing naringenin chalcone formation, the lyase PAL mediating phenylalanine deamination, the isomerase CHI facilitating isomerization, and the ligase 4CL (Table 1). Notably, oxidoreductases dominate this pathway, underscoring the critical role of oxidoreductase reactions in flavonoid biosynthesis.

The flavonoid biosynthetic pathway involves numerous key enzymes that catalyze and regulate flavonoid production (Figure 2). PAL is a rate-limiting enzyme in the phenylpropanoid biosynthesis pathway, controls the synthesis rate of downstream secondary metabolites, including flavonoids [15]. Chalcone synthase (CHS), a pivotal enzyme in the early flavonoid pathway, catalyzes the condensation of *p*-coumaroyl-CoA and malonyl-CoA, followed by intramolecular cyclization to form naringenin chalcone [16]. CHS also serves as the first rate-limiting enzyme in flavonoid biosynthesis, with studies indicating its role in determining flavonoid variation among different radish (*Raphanus sativus*) cultivars [17]. IFS is a key enzyme for isoflavone formation, is predominantly found in *Fabaceae* plants. Although no known IFS homologous gene exists in *Arabidopsis thaliana*, isoflavones are still detected, suggesting an alternative gene may mediate isoflavone skeleton biosynthesis in *Brassicaceae* species [18]. CHI significantly enhances the isomerization efficiency of chalcones to flavanones and acts as a critical rate-limiting enzyme in flavonoid biosynthesis [19]. FNS is the key enzyme responsible for flavone biosynthesis. In plants, flavone production is catalyzed by two distinct flavone synthases, FNS I and FNS II [20]. However, neither of these genes has been identified in *A. thaliana*. FNS II exhibits two catalytic mechanisms: one converts flavanones into the intermediate 2-hydroxyflavanone before yielding flavones, while the other directly synthesizes flavones without intermediates, making it another rate-limiting enzyme [19]. FLS serves as the key enzyme in flavonol biosynthesis, catalyzing the conversion of dihydroflavonols to flavonols. Anthocyanidin synthase (ANS), functioning as both the key and rate-limiting enzyme in anthocyanin production, oxidizes leucoanthocyanidins to anthocyanins, playing crucial roles in plant pigmentation and stress resistance [21]. Recent studies have identified leucoanthocyanidin reductase (LAR) and anthocyanidin reductase (ANR) as the key enzymes responsible for synthesizing flavan-3-ols, the direct precursors of proanthocyanidins. While the biosynthetic pathways of proanthocyanidins have been largely elucidated, the molecular mechanisms governing proanthocyanidin transport, oxidation, and polymerization remain poorly understood and require further investigation.

## 4. Regulation of Flavonoid Biosynthesis

### 4.1. MYB Transcription Factors Regulating Flavonoid Biosynthesis

#### 4.1.1. MYB Family Members Involved in Flavonoid Regulation in *A. thaliana*

Flavonoid biosynthesis in plants is regulated by multiple transcription factors (TFs), among which MYB transcription factors play a pivotal role. Phylogenetic tree analysis revealed that the MYB subfamily involved in the flavonoid metabolic pathway is highly conserved during evolution. Moreover, the evolutionary origin of these subfamilies coincides with the genes encoding key enzymes in the flavonoid biosynthetic pathway, suggesting a co-evolutionary mechanism between MYB genes and flavonoid metabolism. The MYB transcription factor family is defined by the presence of 1–4 repeat domains (R). Based on the structural characteristics of the DNA-binding domain, MYB family transcription factors (TFs) are classified into four subfamilies: 1R-MYB, 2R-MYB, 3R-MYB, and 4R-MYB [22]. Taking *A. thaliana* as an example, MYB TFs involved in the regulation of flavonoid biosynthesis are predominantly R2R3-MYB members, with a minority belonging to the R3-MYB subfamily (Table 2).

**Figure 3:**
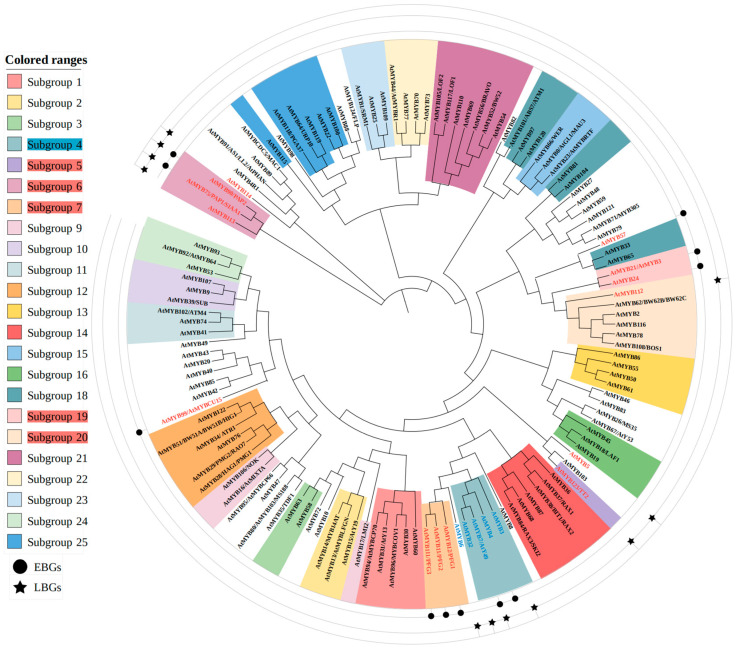
R2R3-MYB family phylogenetic tree in *A. thaliana*. In the figure, R2R3-MYBs labeled in red represent positive regulators of flavonoid biosynthesis in *Arabidopsis*, while those labeled in blue represent negative regulators of flavonoid biosynthesis in *Arabidopsis*. R2R3-MYBs marked with circular symbols regulate early flavonoid biosynthetic genes in *Arabidopsis*, whereas those marked with star symbols regulate late flavonoid biosynthetic genes in *Arabidopsis*.

The R2R3-MYB subfamily represents the predominant class of MYB transcription factors in plants, comprising 126 MYB proteins in *A. thaliana* (Figure 3). R2R3-MYB gene family has undergone significant functional diversification during evolution, resulting in distinct subgroups [23]. The canonical classification and nomenclature of R2R3-MYBs were initially established in *A. thaliana*, where early studies classified them into 25 subgroups [24]. Many of these subgroups function as transcriptional activators or repressors, directly or indirectly regulating the expression of flavonoid biosynthesis genes. However, over 30 R2R3-MYBs remain unclassified into any subgroup [23,24]. Notably, all members of R2R3-MYB Subgroup 5, 6, 7, and 19 are known to positively regulate flavonoid biosynthesis, while AtMYB112 from Subgroup 20 also participates in flavonoid production [25,26,27,28,29,30]. In contrast, R2R3-MYB Subgroup 4 acts as a repressor of flavonoid biosynthesis [31]. Additionally, several unclassified R2R3-MYBs, including AtMYB5, AtMYB6, AtMYB57, AtMYB99, and AtMYB114, have been demonstrated to modulate flavonoid accumulation [27,29,32,33,34].

#### 4.1.2. Positive Regulation of Flavonoid Biosynthetic Genes by MYB Transcription Factors

**Figure 4:**
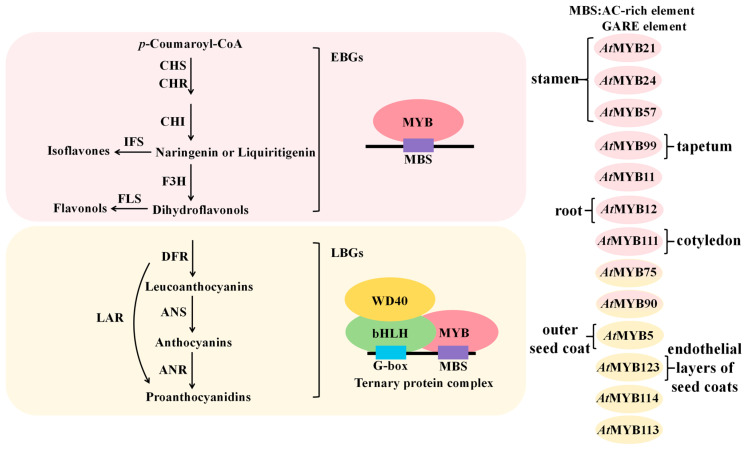
Two major ways in which MYB transcription factors regulate flavonoid synthase genes.

The flavonoid biosynthesis pathway can be divided into two major phases regulated by early biosynthetic genes (EBGs) and late biosynthetic genes (LBGs) (Figure 4). EBGs primarily include genes such as *CHS*, *CHI*, *F3H*, and *FLS*, which are involved in the synthesis of isoflavones, flavones, and flavonols, whereas LBGs mainly consist of *DFR*, *ANR*, and *ANS*, which regulate the production of anthocyanins and proanthocyanidins. Taking *A. thaliana* as an example, MYB transcription factors containing the MYB structural domain can either independently regulate the expression of specific genes in the flavonoid biosynthetic pathway or collaboratively modulate flavonoid biosynthesis by forming the MYB-bHLH-WD40 (MBW) ternary complex with WD40 and bHLH proteins (Figure 4). The highly conserved MYB domain of MYB transcription factors binds to MYB binding sites (MBS) in the promoter regions of target genes. Previous studies have identified that R2R3-MYB transcription factors recognize MBS motifs, including AC-rich elements: 5′-ACC(A/T)A(A/C)-3′ (ACC element) and 5′-(C/T)AACNG-3′ (AAC element), as well as the GARE cis-acting element TTGTTA [29,35,36]. The G-box element CACGTG serves as the binding site for bHLH transcription factors that regulate flavonoid biosynthesis [37].

In *A. thaliana*, members of the R2R3-MYB family AtMYB11, AtMYB12, AtMYB111, AtMYB21, AtMYB24, AtMYB57, AtMYB99, AtMYB75, and AtMYB90 promote flavonoid biosynthesis by directly activating the expression of certain early biosynthetic genes (EBGs) in the flavonoid pathway. Specifically, AtMYB11, AtMYB12, and AtMYB111 recognize and bind to the SMRE element ACC(A/T)A(A/C)(T/C) in the promoters of flavonoid biosynthetic genes, activating the transcription of *FLS*, *CHS*, *CHI*, and *F3H* to enhance flavonol production [25,28,30]. Moreover, studies revealed that the differential regulation of three closely related R2R3-MYB transcription factors (TFs) governs flavonol accumulation in distinct tissues of *A. thaliana* seedlings. Low levels of AtMYB11 transcripts were detected in all seedling samples, whereas AtMYB12 primarily regulates flavonol biosynthesis in roots, and AtMYB111 predominantly controls flavonol biosynthesis in cotyledons [28]. AtMYB21, AtMYB24, and AtMYB57 are homologs that promote flavonol biosynthesis in *A. thaliana* stamens by regulating *FLS1* expression. Among them, AtMYB21 has been demonstrated to transcriptionally activate *FLS1* and enhance flavonol production by binding to the GARE cis-element (TTGTTA) within the *FLS1* promoter [29]. AtMYB99 controls the production of tapetum diglycosylated flavonols, overexpression of *AtMYB99* upregulates the expression of *CHS* and *F3H*, with *CHS* exhibiting the most pronounced increase-over 60-fold higher than in the wild type [32]. Additionally, studies demonstrate that AtMYB75 and AtMYB90 act as transcriptional regulators of early biosynthetic genes (EBGs), including *PAL* and *CHS*, which are essential for anthocyanin production [28].

Studies have demonstrated that the expression of late biosynthetic genes (LBGs) in the anthocyanin and proanthocyanidin biosynthesis pathways requires transcriptional activation by the R2R3-MYB/bHLH/WD40 (MBW) complex [37]. For instance, AtMYB75/AtMYB90/AtMYB113/AtMYB114, GL3/EGL3/TT8 (bHLHs), and TTG1 (WD40) have been confirmed to interact as a ternary complex to coordinately regulate anthocyanin biosynthesis [27]. Furthermore, AtMYB123 (TT2) has been shown to interact with TT8 and TTG1 to modulate proanthocyanidin biosynthesis [27]. Previous studies identified the MBW complex in the endothelial layers of seed coats as being composed of TTG1, TT8, and TT2 [27]. AtMYB5 regulates PA biosynthesis in the outer seed coat, research finds that AtMYB5 physically interacts with TT8, a TTG1-dependent bHLH transcription factor, indicating their functional cooperation in TTG1-mediated developmental pathways [33].

**Figure 5:**
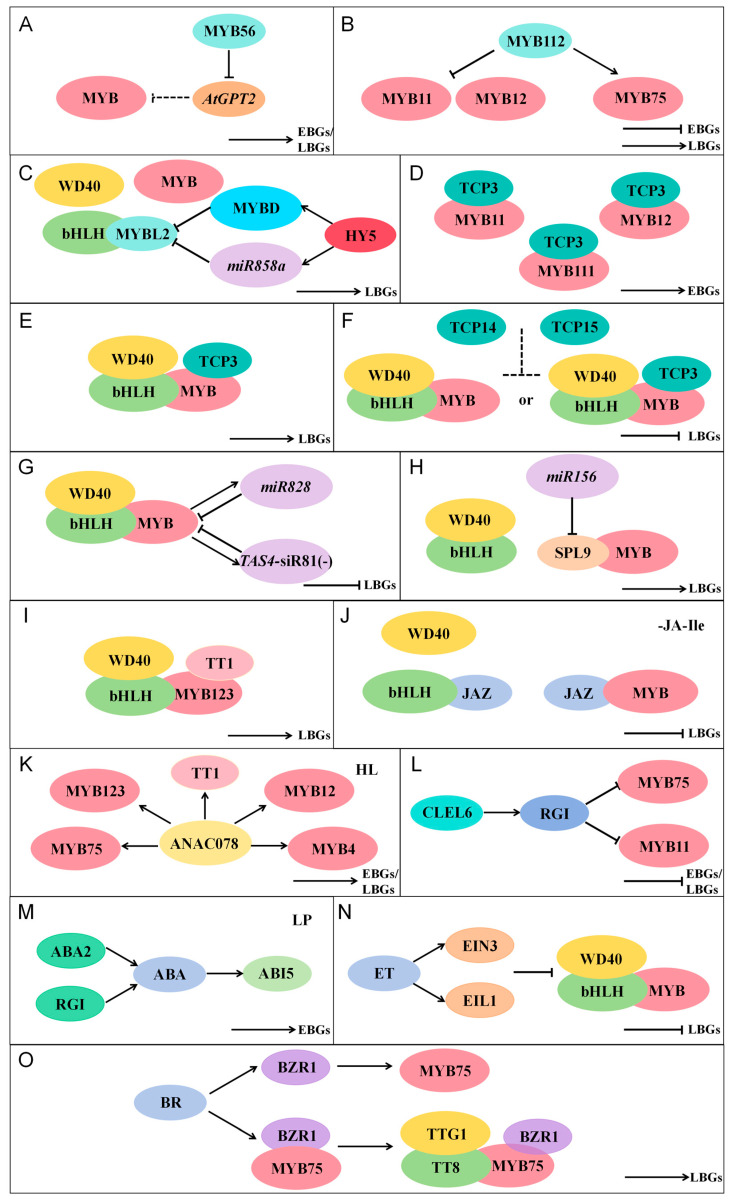
Alternative ways of regulating flavonoid biosynthetic pathways. (**A**) AtMYB56 regulates flavonoid biosynthesis by modulating AtGPT2; (**B**) AtMYB112 promotes anthocyanin biosynthesis by activating PAP1, while suppresses flavonol synthesis through inhibiting AtMYB12 and AtMYB111; (**C**) The regulatory control systems of AtHY5-AtMYBD-AtMYBL2 and AtHY5-miR858a-AtMYBL2 for promoting anthocyanin biosynthesis; (**D**) TCP3 interacts with AtMYB11, AtMYB12, and AtMYB111 to form binary complexes that promote flavonol biosynthesis; (**E**) TCP3 promotes flavonoid biosynthesis by stabilizing the MBW ternary complex; (**F**) TCP14 and TCP15 indirectly regulate flavonoid biosynthesis; (**G**) MiR828 and *TAS4*-siRNA81(-) reduce anthocyanin biosynthesis by targeting MYB transcription factors; (**H**) MiR156 negatively regulates anthocyanin accumulation by targeting SPL9 to disrupt the MBW ternary complex; (**I**) TT1 interacts with AtMYB123 to promote PAs accumulation; (**J**) In the absence of JA-Ile, JAZ proteins inhibit anthocyanin biosynthesis by interfering with the interaction between bHLHs and AtMYB75; (**K**) Under HL conditions, ANAC078 promotes flavonoid biosynthesis by regulating AtMYB75, AtMYB123, TT1, AtMYB12, and AtMYB4; (**L**) CLEL6 inhibits anthocyanin biosynthesis by regulating AtMYB75 and AtMYB11 through RGI receptors; (**M**) Under LP conditions, ABA promotes flavonoid biosynthesis through the transcription factor ABI5; (**N**) ET suppresses anthocyanin accumulation by acting on the MBW complex through transcription factors EIN3 and EIL1; (**O**) BR promotes flavonoid biosynthesis by regulating AtMYB75 and the MBW complex through transcription factor BZR1. In the figure, arrows indicate promotion, hammerheads indicate inhibition, and dashed lines indicate indirect regulation.

In addition, sugars function as signaling molecules whose transduction pathways can activate or repress gene expression. Studies demonstrate that sucrose (Suc) treatment strongly upregulates both flavonoid and anthocyanin biosynthetic pathways [38]. Initial investigations on the effects of sugars (Suc, glucose, and fructose) on flavonoid biosynthetic enzyme-encoding genes in *A. thaliana* demonstrated that the sugar-dependent upregulation of anthocyanin biosynthesis is specifically induced by sucrose [38]. The sucrose signaling pathway modulates the accumulation of both flavonoids and anthocyanins in *A. thaliana*. AtMYB56 mediates this regulation by controlling *AtGPT2* expression in response to sucrose, thereby influencing anthocyanin levels (Figure 5). Although AtMYB56 promotes the accumulation of both anthocyanins and flavonols, the reduced anthocyanin content observed in *myb56* mutants appears independent of changes in anthocyanin biosynthetic gene expression, because AtMYB56 functions as a key regulator of sucrose-induced *AtGPT2* expression in a circadian-dependent manner, ultimately altering cellular free maltose levels and consequently affecting nuclear anthocyanin accumulation [39]. AtMYB112 exhibits broad expression across most tissues during seedling and plant development, with particularly high expression in leaves and pollen [26]. AtMYB112 functions as a dual-specificity transcriptional regulator, activating *PAP1* to promote anthocyanin biosynthesis while simultaneously repressing *AtMYB12* and *AtMYB111* to inhibit flavonol production, thereby serving as both a positive regulator of anthocyanins and a negative regulator of flavonols [26].

#### 4.1.3. Negative Regulation of Flavonoid Biosynthetic Genes by MYB Transcription Factors

Certain MYB transcription factors (TFs) function as negative regulators of flavonoid biosynthesis in plants. Most flavonoid-repressing MYB TFs belong to the R2R3-MYB subgroup 4 or its homologs, with only a few members from the R3-MYB family. Within R2R3-MYB subgroup 4, AtMYB3, AtMYB4, AtMYB7, and AtMYB32, along with AtMYB6 (which shares high sequence similarity with subgroup 4 proteins), act as transcriptional repressors in the flavonoid pathway. Studies demonstrate that elevated expression of transcriptional repressors *AtMYB3*, *AtMYB6*, and *AtMYBL2* coupled with reduced expression of key *bHLHs* (*TT8*, *TTG1*, and *EGL3*) correlates with downregulated anthocyanin biosynthesis in leaves under high-temperature and low-light (HTLL) conditions [34]. The four closely related subgroup 4 TFs (AtMYB3/4/7/32) are predicted to interact with bHLH proteins to mediate transcriptional repression of flavonoid biosynthesis [31]. Overexpression of *AtMYB4* was demonstrated to suppress the transcription of *C4H*, *4CL*, and *CHS* genes [40], while AtMYB7 negatively regulates flavonol biosynthesis by downregulating the expression of *CHI*, *FLS*, and *DFR* [41]. Additionally, AtMYB32 represses anthocyanin accumulation by reducing the transcript levels of *DFR* and *ANS* [42]. *AtMYB4* is detectable in both filaments and styles, with ubiquitous expression throughout leaf tissues [41]. *AtMYB7* exhibits predominant expression in roots and anthers, where it primarily modulates flavonoid biosynthesis in the leaf vasculature of rosettes [42]. *AtMYB32* displays broad tissue distribution but shows the strongest expression in the anther tapetum, stigma papillae, and lateral root primordia [42].

Additionally, certain MYB transcription factors (TFs) do not directly regulate flavonoid biosynthetic enzyme genes but instead modulate flavonoid accumulation through competitive binding or indirect regulation of associated factors (Figure 5). For instance, AtMYBL2 unlike other small R3-MYBs-does not function redundantly and is the sole small R3-MYB protein known to interfere with the flavonoid pathway. It negatively regulates anthocyanin biosynthesis by suppressing the activity of the MBW ternary complex [43,44,45]. Mechanistically, AtMYBL2 disrupts MBW complex formation by competitively binding to bHLH transcription factors, thereby inhibiting the transcription of late biosynthetic genes (LBGs) and reducing anthocyanin production. Studies demonstrate that loss of *AtMYBL2* function in *mybl2* knockout mutants does not affect flavonol or proanthocyanidin (PA) biosynthesis in seeds or vegetative tissues. However, it leads to hyperaccumulation of anthocyanins, accompanied by upregulated expression of both structural and regulatory (*DFR*, *LDOX*, *GL3*, *TT8*, *PAP1*) genes involved in anthocyanin synthesis [43,44].

### 4.2. Additional Factors Regulating Flavonoid Biosynthesis

#### 4.2.1. Regulation of Flavonoid Biosynthesis by Other TFs

Studies have revealed that multiple additional transcription factors (TFs) participate in the flavonoid biosynthetic pathway by directly interacting with MYB factors or the MBW complex (Figure 5). These include TCP proteins (TCP3, TCP15) from the TCP family, NAC protein (ANAC078), SQUAMOSA PROMOTER BINDING PROTEIN-LIKE9 (SPL9) and a WIP-type zinc finger protein (TT1) belonging to subgroup A1d of *A. thaliana* zinc finger proteins [45,46,47,48,49].

TCP proteins belong to the plant-specific bHLH transcription factor family and are key regulators of a variety of developmental processes [47]. TCP proteins regulate diverse biological processes in plants, based on variations in their plant-specific TCP structural domains can be classified into two major types, Class I (TCP-P) and Class II (TCP-C), Class II is further subdivided into CIN and CYC/TB1 subgroups [50]. Class I TCP protein TCP15 acts as a transcriptional repressor of anthocyanin accumulation. Its regulatory role appears indirect, potentially mediated through modulation of *AtMYB75* and other anthocyanin regulatory genes [46]. Mechanistically, TCP15 may reduce anthocyanin levels by destabilizing the MBW complex or interfering with TCP3-complex interactions. In contrast, the Class II CIN-TCP protein TCP3 promotes flavonoid biosynthesis by forming binary complexes with R2R3-MYB proteins [47]. For instance, TCP3 interacts with AtMYB11, AtMYB12, and AtMYB111 to enhance transcription of early biosynthetic genes (EBGs), thereby increasing flavonol production [45]. TCP3 also associates with anthocyanin-specific MYBs (AtMYB75/90/113/114) and the proanthocyanidin regulator AtMYB123, indicating its additional role in regulating late biosynthetic genes (LBGs) [47]. Furthermore, TCP3 stabilizes MBW complex formation to activate LBG transcription and promote flavonoid expression [45].

The NAC family represents one of the largest transcription factor families in plant genomes and has been demonstrated to be critical regulators of abiotic stress responses, with 106 predicted members in *A. thaliana* [49,51]. In *A. thaliana*, overexpression of *NAC* genes enhances plant stress tolerance. For instance, the ANAC078 protein in *A. thaliana* is associated with the expression of flavonoid biosynthesis-related genes, leading to anthocyanin accumulation under high light (HL) stress [49]. Study findings revealed that under high light (HL) stress, transcription factors regulating flavonoid biosynthesis-related gene expression were upregulated in *ANAC078*-overexpressing (Ox-*ANAC078*) plants, including *PAP1*, *TT1*, *TT2*, *AtMYB12*, and *AtMYB4* [49]. However, the transcription factors controlling ANAC078 expression remain unidentified [49]. The study further analyzed anthocyanin levels in wild-type (WT), Ox-*ANAC078*, and KO-*ANAC078* plants under HL stress [49]. After 2 days of HL treatment, anthocyanin content increased in WT, Ox-*ANAC078*, and KO-*ANAC078* plants, with Ox-*ANAC078* exhibiting significantly higher anthocyanin accumulation than WT. In contrast, KO-*ANAC078* plants showed lower anthocyanin levels compared to WT [49].

SPL9 functions as a negative fine-tuner of anthocyanin biosynthesis by competitively disrupting MBW complex formation through interference with R2R3-MYB and bHLH interactions, thereby suppressing anthocyanin accumulation [45]. Studies reveal that at least one miR156/157-targeted SPL9 competes with TT8 for binding to anthocyanin-related R2R3-MYBs (AtMYB75 and AtMYB113), consequently destabilizing the MBW complex. This leads to direct transcriptional repression of key anthocyanin biosynthetic genes, including *F3’H*, *DFR*, *BAN*, and *UGT75C1* [48].

TT1 encodes a WIP-type zinc finger protein that, along with five other WIP proteins, belongs to subgroup A1d of *A. thaliana* zinc finger proteins. Functioning as a positive fine-tuner of proanthocyanidin (PA) biosynthesis in the endothelial cells of the seed coat, TT1 interacts with AtMYB123 to positively regulate PA accumulation in these specialized cells [45].

#### 4.2.2. Regulation of Flavonoid Biosynthesis by miRNAs

miRNAs are 20–24 nt noncoding endogenous small RNAs, accumulating evidence indicates that miRNAs and other small RNAs can regulate the expression levels of key factors involved in anthocyanin biosynthesis (Figure 5) [52,53]. In *A. thaliana*, miR156 modulates the biosynthesis of flavonoids. Increased miR156 activity promotes anthocyanin accumulation, whereas reduced miR156 activity leads to elevated flavonol levels [48]. Further studies demonstrated that at least one miR156 target, SPL9, directly suppresses the expression of anthocyanin biosynthetic genes by disrupting the MYB-bHLH-WD40 transcriptional activation complex, thereby negatively regulating anthocyanin accumulation [48]. *Trans-acting siRNA* (*TAS*) genes are loci that generate small interfering RNAs (siRNAs), which regulate target gene expression in trans [54]. In *A. thaliana*, miR828 triggers the cleavage of *Trans-acting siRNA Gene 4* (*TAS4*) transcripts, producing small interfering RNAs (siRNAs), known as *ta*-siRNAs [54]. miR828 and one siRNAs, *TAS4*-siRNA81(−), regulate the anthocyanin biosynthetic pathway by targeting MYB transcription factors (TFs) [54]. Overexpression of *miR828* reduces the expression levels of MYB TFs, thereby decreasing anthocyanin accumulation. Conversely, increased *TAS4* expression generates more *TAS4*-siR81(−) through miR828-mediated cleavage, which subsequently downregulates MYB transcript levels by targeting other genes, including MYB TFs [53,54].

Recent studies have revealed that miR858a participates in anthocyanin regulatory control systems, AtHY5-AtMYBD-AtMYBL2 and AtHY5-miR858a-AtMYBL2, which function in flavonoid biosynthesis [55]. The translation of AtMYBL2 can be suppressed by miR858a, while AtMYBD inhibits *AtMYBL2* expression by directly binding to its promoter, thereby promoting anthocyanin biosynthesis. Additionally, *AtMYBL2* gene expression is negatively regulated by ELONGATED HYPOCOTYL5 (HY5). Consequently, the AtHY5-AtMYBD-AtMYBL2 and AtHY5-miR858a-AtMYBL2 pathways collectively form an integrated control system for anthocyanin regulation [55].

#### 4.2.3. Regulation of Flavonoid Biosynthesis by Plant Hormones

The plant hormone jasmonic acid (JA), along with its cyclic precursors and derivatives, has been demonstrated to upregulate the expression of key enzymatic genes involved in the anthocyanin biosynthetic pathway (Figure 5). Among bioactive JAs, jasmonoyl-L-isoleucine (JA-Ile) plays a crucial role by mediating the degradation of JAZ (Jasmonate ZIM-domain) transcriptional repressors [37]. In the absence of JA-Ile, JAZ proteins form (hetero) dimers that interact with bHLH transcription factors and AtMYB75, maintaining these regulators in an inactive state, this interaction disrupts the AtMYB75-bHLH transcriptional complex, thereby suppressing the expression of anthocyanin biosynthetic genes [37].

Low concentrations of gibberellic acid (GA_3_) have been shown to promote the expression of genes in the anthocyanin biosynthetic pathway. However, studies demonstrate that gibberellins (GAs) generally suppress sucrose-induced anthocyanin biosynthesis. For instance, in the *A. thaliana ga1-5* mutant, reduced endogenous GA levels lead to upregulation of key anthocyanin biosynthetic genes such as *CHI* and *CHS*, resulting in enhanced anthocyanin accumulation [56].

Abscisic acid (ABA) plays a pivotal role in regulating plant responses to various adverse environmental stimuli. Studies have revealed that ABA is essential for low phosphate (LP)-induced anthocyanin accumulation in *A. thaliana*, where ABA participates in LP-triggered anthocyanin biosynthesis through the transcription factor ABA Insensitive5 (ABI5) [57]. Phosphate (Pi) deficiency upregulates the expression of *ABA DEFICIENT2* (*ABA2*), a key ABA-biosynthetic gene, and *BETA-GLUCOSIDASE1* (*BG1*), a major gene implicated in converting conjugated ABA to active ABA, leading to increased ABA accumulation and subsequent activation of the ABA signaling pathway. ABI5, a core transcription factor involved in ABA signaling, functions downstream of the ABA pathway. Under LP conditions, ABI5 binds to and activates *CHS*, positively regulating the expression of anthocyanin biosynthetic genes, thereby promoting anthocyanin accumulation in plants under LP stress [57].

Research has demonstrated that in *A. thaliana*, the ethylene (ET)-stabilized transcription factors ETHYLENE-INSENSITIVE3 (EIN3) and EIN3-LIKE1 (EIL1) interact with the MBW complex to suppress both the interactions among its bHLH-MYB components and their transcriptional activity, thereby inhibiting JA-induced anthocyanin accumulation [58]. EIN3 and EIL1 can bind to multiple components of the MBW complex, including GL3, ENHANCER OF GLABRA3 (EGL3), TT8, GL1, AtMYB75, and TRANSPARENT TESTA GLABRA1 (TTG1). This binding interferes with either the assembly or transcriptional activity of bHLH-MYB pairs (TT8-AtMYB75, EGL3-AtMYB75, GL3-GL1, and EGL3-GL1), leading to downregulation of *DFR* expression and consequently impairing anthocyanin accumulation [58]. Furthermore, studies have revealed that ethylene also represses sucrose-induced anthocyanin accumulation by downregulating the expression of the sucrose transporter SUC1 [59].

Brassinosteroids (BRs) are a class of plant steroid hormones that play crucial roles in various physiological and developmental processes, including cell growth, photomorphogenesis, stomatal movement and development, vascular differentiation, and reproductive organ development [59]. Studies have revealed that brassinosteroid (BR)-induced anthocyanin accumulation in *A. thaliana* leaves is mediated through dual regulation of PAP1 by the responsive transcription factor BRASSINAZOLE RESISTANT1 (BZR1) in *A. thaliana* seedlings [59]. BZR1 can directly bind to the promoters of PAP1 and PAP2 both in vitro and in vivo, while physically interacting with the PAP1 protein to coregulate the expression of PAP1 target genes, including *TRANSPARENT TESTA8* (*TT8*), *DIHYDROFLAVONOL 4-REDUCTASE*, and *LEUKOANTHOCYANIDIN DIOXYGENASE* (*LDOX*), thereby promoting the expression of anthocyanin biosynthetic genes [59]. Furthermore, BZR1 enhances the transcriptional activity of the MBW protein complex, and studies demonstrate that BZR1 forms protein complexes with PAP1, TT8, and TTG1 in plant cells [59]. Additionally, BZR1-mediated regulation of PAP1 is essential for low nitrogen (N)-induced anthocyanin accumulation. Under low N conditions, the expression of both *PAP1* and *BZR1* is induced, facilitating their functional interaction to enhance anthocyanin biosynthesis [59]. Beyond its dependence on BZR1, BRs can also induce anthocyanin accumulation by upregulating anthocyanin biosynthetic gene expression. Research shows that brassinolide (BL, the most active BR) significantly promotes the expression of anthocyanin biosynthetic genes (*CHS*, *CHI*, *F3H*, *F3′H*, *DFR*, *LDOX*, and *UF3GT*). Notably, BL also increases the expression of key transcription factors (*PAP1*, *PAP2*, and *TT8*) that upregulate anthocyanin biosynthetic genes [59].

#### 4.2.4. Regulation of Flavonoid Biosynthesis by Sulfopeptide

The posttranslationally modified sulfopeptide CLE-LIKE6 (CLEL6) acts as a negative regulator of flavonoid biosynthesis in etiolated and light-stressed *A. thaliana* seedlings [60]. Studies revealed that overexpression and exogenous application of *CLEL6* (DYPQPHRKPPIHNE) suppressed the expression of anthocyanin biosynthesis genes in etiolated and light-stressed seedlings, confirming its role as an inhibitor of anthocyanin biosynthesis. Conversely, when *CLEL6* expression was downregulated, anthocyanin biosynthesis was derepressed [60]. The study demonstrated that *CLEL6* overexpression or application suppresses anthocyanin biosynthesis through ROOT MERISTEM GROWTH FACTOR 1 INSENSITIVE (RGI) receptor(s). Mature CLEL6 activates RGI receptor(s), and the active RGI signaling pathway subsequently inhibits anthocyanin synthesis by downregulating the expression of the transcription factors AtMYB75 and AtMYB11 [60].

## 5. Competitive Relationships in Biosynthesis Among Different Flavonoids

### 5.1. Competition Between Anthocyanin and Flavonol Biosynthesis

**Figure 6:**
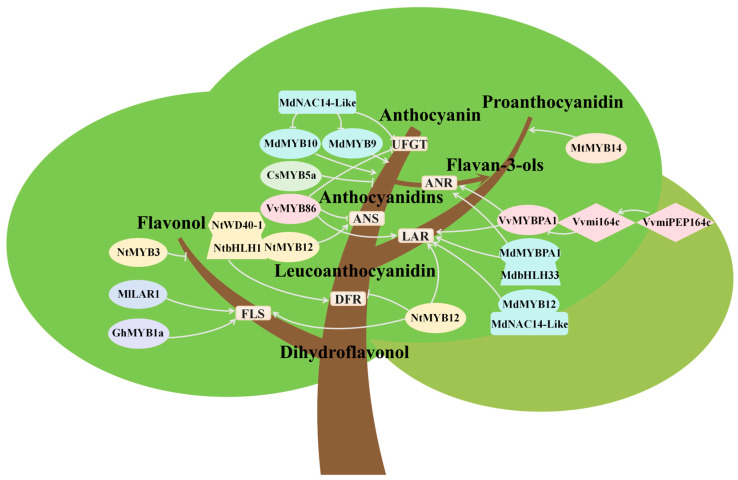
Competition for biosynthesis among different flavonoids. In the flavonoid biosynthesis pathway, anthocyanins and flavonols, as well as anthocyanins and proanthocyanidins, compete for the same substrates through the regulation of enzyme activities and/or biosynthetic gene expression by various transcription factors. In the figure, different shapes and colors represent distinct transcription factors and species, where arrows indicate promotion and hammerheads indicate inhibition.

Anthocyanins and flavonols play crucial roles in plant development and defense. Since they share common biosynthetic precursors, competitive interactions occur between the anthocyanin and flavonol biosynthesis pathways in many plant species [61,62]. Dihydroflavonols serve as key intermediates and a metabolic branch point for both anthocyanin and flavonol biosynthesis, they are essential substrates for the production of these flavonoids and simultaneously represent the competitive node between the two pathways (Figure 6).

Competition between anthocyanin and flavonol biosynthesis is widely observed across plant species. For instance, comparative analysis of ten *Rhododendron* species revealed an inverse correlation between anthocyanin and flavonol content in petals, with flavonol-rich species exhibiting low anthocyanin accumulation [63]. In *Malus*, the metabolic competition between anthocyanins and kaempferol influences pollen tube growth and seed set rates [64]. This competitive relationship is primarily regulated by key biosynthetic enzymes, particularly *DFRs* and *FLSs* [64]. Studies demonstrate that in *Mimulus lewisii*, the R2R3-MYB transcription factor *LIGHT AREAS1* (*MlLAR1*) positively regulates *FLS* expression, redirecting metabolic flux of dihydroflavonols from the anthocyanin biosynthetic pathway toward flavonol production, this consequently suppresses anthocyanin biosynthesis in the white region (light areas) surrounding the corolla throat [65]. In *Solanum lycopersicum*, *DFR*-deficient anthocyaninless mutants exhibit elevated flavonol accumulation concomitant with impaired anthocyanin biosynthesis [66]. Constitutive overexpression of *FLS* genes derived from *Rosa rugosa*, *Prunus persica*, and *Petunia hybrida* in tobacco resulted in white flowers, whereas heterologous overexpression of *DFR* led to enhanced accumulation of anthocyanins, producing redder floral coloration [67]. Studies reported that heterologous expression of *MaFLS* from *Muscari aucheri* in *Nicotiana tabacum* upregulated *NtFLS* expression but suppressed key anthocyanin biosynthetic genes (including *NtDFR*, *NtANS*, and *NtAN2*), thereby reducing pigmentation in tobacco petals [61]. Similarly, overexpression of the onion *FLS* gene (*AcFLS-HRB*) increased flavonol content while decreasing anthocyanin accumulation in tobacco petals, resulting in pale pink flowers [61].

In addition to structural genes, the biosynthesis of flavones is regulated by numerous transcription factors (TFs). Evidence indicates that MYBs play distinct roles in modulating the biosynthesis of flavonols and anthocyanins. For instance, overexpression of the *Malus MdMYB10* enhanced anthocyanin accumulation in flowers but reduced the content of kaempferol 3-O-glycosides [64]. In *Narcissus tazetta*, NtMYB12 interacts with NtbHLH1 and NtWD40-1 to form a MYB-bHLH-WD40 triplex, specifically activating the expression of *NtDFR* and *NtANS* to promote (pro)anthocyanin accumulation. In contrast, NtMYB12 alone activates *NtFLS* and *NtLAR* expression, leading to flavonol accumulation while suppressing *NtDFR* expression [68]. These findings demonstrate that NtMYB12 either alone or as part of the NtMYB12-bHLH1-WD40-1 triplex mediates metabolic competition between flavonol and (pro)anthocyanin biosynthesis [68]. A detailed characterization of the R2R3-MYB transcription factor GhMYB1a from *Gerbera hybrida* revealed its role as a regulator of flavonol biosynthesis. Overexpression of *GhMYB1a* primarily influenced flavonol accumulation and reduced anthocyanin levels by directly upregulating early flavonoid pathway genes (*CHS*, *F3H*, *FLS*) without directly affecting late genes (*F3′H*, *DFR*, *ANS*) [62]. The inverse correlation between anthocyanin and flavonol levels in *GhMYB1a*-overexpressing lines reflects competition between these two flavonoid branches [62]. In *Narcissus tazetta* var. *chinensis*, NtMYB3 induced PA accumulation by suppressing flavonol biosynthesis [69]. Furthermore, heterologous expression of subgroup 7 MYB transcription factors in transgenic plants can modulate flavonoid pathway gene expression, particularly by upregulating flavonol biosynthetic genes, thereby enhancing flavonol production while inhibiting anthocyanin accumulation [70].

### 5.2. Competition Between Anthocyanin and Proanthocyanidin Biosynthesis

Anthocyanins are natural water-soluble pigments that are widely distributed in various tissues and organs of higher plants, providing vibrant colors and playing critical roles in physiological processes related to plant growth and development [71]. Proanthocyanidins (PAs), another class of flavonoids, primarily function as a defense mechanism against a broad range of biotic and abiotic stresses, exhibiting diverse biological activities and beneficial functions [71]. In plants, the biosynthesis of anthocyanins and PAs shares a common early pathway, both originating from the substrate phenylalanine, which undergoes enzymatic catalysis to produce leucoanthocyanidins. Subsequently, leucoanthocyanidins serve as the first metabolic branch point in both anthocyanin and PA biosynthetic pathways, under catalysis by LAR, they are converted to (+)-catechins, while ANS mediates their transformation into anthocyanidins. The resultant anthocyanidins can be further modified by ANR to yield (−)-epicatechins. The condensation of (+)-catechins and (−)-epicatechins ultimately generates proanthocyanidin polymers. Furthermore, anthocyanidins possess glycosylation potential and can be stabilized through the activity of UDP-glucose: flavonoid-3-O-glucosyltransferase (UFGT), forming more stable anthocyanin glycosides [72]. Consequently, metabolic competition exists between the anthocyanin and PA biosynthetic pathways for two key substrates: leucoanthocyanidins and unstable anthocyanidins (Figure 6).

This competitive and interconversion process is endogenously regulated in plants to modulate anthocyanin and PA biosynthesis, enabling adaptation to varying physiological and environmental conditions [73]. For instance, a micropeptide (miPEP) miPEP164c/miPEP-MYBPA1 encoded within immature *miR164c* was identified as potentially targeting the transcription factor VvMYBPA1 in *Vitis vinifera*. This regulatory element acts as a positive regulator of key PA biosynthetic genes while competing directly with the anthocyanin biosynthesis pathway for substrates [73]. Further investigation revealed that miPEP164c predominantly suppresses the PA pathway through inhibition of LAR and ANR enzymatic activities, coupled with downregulation of *VvLAR1*, while simultaneously stimulating anthocyanin biosynthesis [73]. Additionally, studies have demonstrated that *MdMYBPA1* in red-fleshed apple (*Malus sieversii* f. *niedzwetzkyana*) redirects flavonoid biosynthesis from PAs to anthocyanin production in response to low temperatures [74]. Through yeast one-hybrid assays, electrophoretic mobility shift assays (EMSAs), and luciferase reporter assays in red-fleshed apples, the NAC transcription factor MdNAC14-Like was shown to bind promoters of *MdMYB9*, *MdMYB10*, and *MdUFGT*, thereby suppressing their transcriptional activity and subsequent anthocyanin synthesis. Moreover, MdNAC14-Like interacts with MdMYB12 to enhance its transcriptional activation of the downstream structural gene *MdLAR*, promoting PA biosynthesis [71]. A recent study identified an R2R3-MYB transcription factor, VviMYB86, whose spatiotemporal expression pattern in grape berries positively correlates with proanthocyanidin (PA) accumulation but negatively correlates with anthocyanin biosynthesis [72]. Both in vivo and in vitro experiments demonstrated that VviMYB86 primarily functions in *A. thaliana* protoplast systems by upregulating two *LAR* genes to promote PA biosynthesis. Furthermore, VviMYB86 suppresses the anthocyanin biosynthetic branch in grapes by downregulating transcript levels of *VviANS* and *VviUFGT* [72]. Recent studies have identified additional MYB transcription factors (TFs) that differentially regulate distinct branches of the flavonoid biosynthetic pathway in various plant species [72]. In *Medicago truncatula* hairy roots overexpressing MtMYB14, proanthocyanidin (PA) accumulation was significantly enhanced, while anthocyanin production was reduced by approximately 50% [75]. Transgenic *Nicotiana tabacum* plants overexpressing the *Camellia sinensis CsMYB5a* gene exhibited significantly reduced anthocyanin accumulation, while demonstrating elevated levels of polymeric proanthocyanidins (PAs) in floral tissues [76].

## 6. Environmental Factors Affecting Flavonoid Biosynthesis

### 6.1. Biological Factors

**Figure 7:**
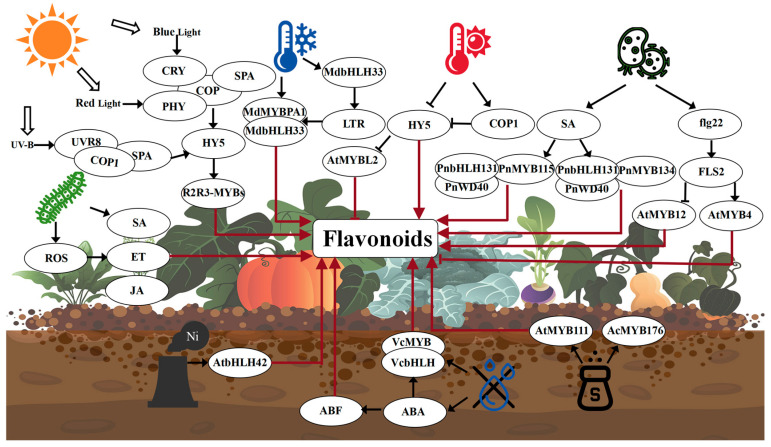
Environmental factors affecting flavonoid synthesis. In the figure, different icons represent environmental factors affecting flavonoid biosynthesis, including light, temperature, water availability, pests/diseases, salinity stress, and heavy metals. The arrows indicate promotion, while the hammerheads indicate inhibition.

Under biotic stresses such as pathogen or insect pest infestation, the phenylpropane metabolic synthesis pathway becomes highly active in many plants, providing precursor substances for the biosynthesis of flavonoids, monolignols, and phenolics (Figure 7). Flavonoids have been extensively evaluated due to their antibacterial properties, including inhibitory effects on various pathogenic microorganisms (including multidrug-resistant bacteria) [77]. Studies indicate that flavonoids exhibit strong antimicrobial activity (antibacterial, antifungal, and antiviral), prompting their synthesis in plants upon pathogen attack as a defense mechanism against biotic stresses [78]. For instance, inoculation with *Xanthomonas campestris* pv. *campestris* significantly increased cell wall-bound phenolic metabolites in the resistant rapeseed cultivar *Brassica napus*, accompanied by elevated jasmonic acid levels and upregulation of genes in the phenylpropanoid biosynthetic pathway [79]. Additionally, the study revealed that in leaves of *Populus nigra* infected by the biotrophic rust fungus *Melampsora larici-populina*, salicylic acid (SA) biosynthesis was upregulated and subsequently activated downstream signaling pathways. SA induced the transcription of PnMYB134, PnMYB115, PnbHLH131, and PnWD40, which substantially upregulated genes involved in flavan-3-ol biosynthesis and enhanced the accumulation of catechin and PAs, ultimately exerting a negative impact on rust colonization [80].

Flavonoid compounds not only play a critical role in response to pathogenic microorganisms but also react to insect pest infestations. Previous studies have demonstrated that oviposition by *Pieris brassicae* induces flavonol accumulation, while infestation by the predatory mite *Tetranychus urticae* upregulates genes associated with flavonoid biosynthesis [81]. Concurrently, studies revealed that the response and regulation of flavonoid biosynthesis vary depending on the pest species. For instance, in leaves infested by *P. brassicae*, differentially expressed genes (DEGs) associated with flavonoid biosynthesis and regulation were upregulated, whereas *T. urticae*-infested leaves exhibited relatively limited differential expression [81]. Notably, in roots of *P. brassicae*-infested plants, the expression levels of genes involved in flavonoid compound synthesis were significantly increased. This herbivore-induced upregulation of flavonoid production pathways in roots may help mitigate possible imbalances caused by insect herbivory, thereby enabling flavonoids to serve as mediators for stress response, antioxidant activity, or root interactions with other organisms [81]. Moreover, studies have demonstrated that flavonoid compounds are induced during nematode infection in plant roots, with more pronounced accumulation in resistant plant cultivars compared to susceptible cultivars [82]. Research has revealed that both the plant host and plant-parasitic nematodes (PPNs) can directly or indirectly manipulate the flavonoid biosynthesis pathway during PPN pathogenesis. For instance, yellow-coloured cyst nematodes are capable of converting quercetin and kaempferol into a unique flavonoid compound-quercetagetin [82]. The flavonoid biosynthesis pathway in plants can be induced by a broad spectrum of pathogen responses through the crosstalk of jasmonic acid, salicylic acid, ethylene, auxin, and reactive oxygen species (ROS), which is likely triggered by mechanical damage and wounding caused during PPNs feeding and penetration processes [82].

However, under biotic stresses, while certain flavonoids with antimicrobial activity and signaling molecule functions, such as catechins and quercetin are induced, the overall flavonoid biosynthesis pathway is constrained. This is because phenylpropane metabolites are preferentially channeled into lignin synthesis to reinforce physical barriers against pathogen invasion [83]. For instance, studies in *A. thaliana* have revealed that crosstalk between the abiotic stress (UV-B) and biotic stress (flg22) suppresses flavonol production. This crosstalk involves the antagonistic regulation of two opposing MYB transcription factors: the flavonol pathway’s positive regulator MYB12 (UV-B-induced but flg22-suppressed) and negative regulator MYB4 (induced by both UV-B and flg22) [83].

### 6.2. Abiotic Factors

Light is an essential environmental factor for plant growth and development, including light quality and light intensity. Different light qualities with varying energy levels significantly influence plant growth and development, thereby affecting the synthesis and accumulation of flavonoids in plants (Figure 7). Studies have shown that flavonoid biosynthesis-related genes, such as *PAL*, *CHS*, and *F3H*, can respond to UV-B, UV-A, and blue light (BL) through the mediation of several photoreceptors [84]. For example, UV-B-activated UVR8 monomers directly interact with the positive regulator CONSTITUTIVE PHOTOMORPHOGENIC1 (COP1), stabilizing ELONGATED HYPOCOTYL5 (HY5) and enhancing HY5 binding to the promoter regions of UV-B-responsive genes [85]. MYB12, as a UV-B-induced FLS specific positive regulator, together with *LAR* and *ANR2* transcriptional repressors MYB4, transmits the UV-B response in early UVR8 mediated signaling events to relevant genes in the flavonoid biosynthetic pathway, ultimately regulating the accumulation of flavonols and catechins in plants [85]. BL-triggered CRY2/3-COP1-SPA1 interaction mediates photomorphogenesis, promoting the binding of transcription factors (TFs) such as HY5 and R2R3-MYBs to downstream flavonoid biosynthesis genes (e.g., *CHI*, *CHS*, and *FLS*). For instance, studies have shown that BL induces the expression of *CRY2/3*, *SPAs* (*SUPPRESSOR OF PHYA*), *HY5*, and *R2R3-MYBs*, thereby enhancing the accumulation of anthocyanins and catechins in tea leaves [86,87]. Additionally, blue shade treatment can relatively enhance the biosynthesis of flavonol glycosides in fresh tea leaves, supplemental blue LED light treatment upregulates flavonoid biosynthesis in tea young shoots [84]. High light intensity promotes flavonoid accumulation, whereas shade treatments largely suppress flavonoid biosynthesis. Under light conditions, the COP1/SUPPRESSOR OF PHYA (SPA) complex interacts with activated photoreceptors PHY and CRY, leading to the dissociation and nuclear export of COP1, thereby inhibiting COP1/SPA function [88]. The low abundance of COP1 in the nucleus facilitates the accumulation of nuclear-localized transcription factors and induces the expression of related genes [88]. HY5, a direct target of COP1, is a bZIP transcription factor that promotes photomorphogenesis, when COP1 is removed from the nucleus, HY5 becomes stabilized under light, activating flavonoid synthesis-related TFs (e.g., R2R3-MYBs) and key structural genes responsible for light-induced accumulation, thereby enhancing flavonoid biosynthesis [88]. Since nuclear export of COP1 occurs relatively slowly, transcription factors can only accumulate under prolonged light exposure, consequently, increased illumination promotes flavonoid biosynthesis in plants [88]. For example, under light intensity stress, the bHLH transcription factor in *Syringa oblata* is significantly upregulated, potentially regulating the flavonoid biosynthesis pathway [89]. Conversely, shade treatments reduce flavonoid biosynthesis in plants. In tea plants (*Camellia sinensis*), the decline in flavonols and catechins under shading is primarily attributed to reduced UV-B radiation, leading to the downregulation of biosynthetic genes and flavonoid-related TFs [85]. The decreased biosynthesis of flavonol glycosides in shaded tea leaves may result from the downregulated expression of upstream genes (*PAL*, *CHS*, *F3H*, and *F3′H*) [84].

Water is a crucial environmental factor influencing plant growth and development. When subjected to abiotic stresses, plants accumulate substantial amounts of flavonoids (Figure 7). Consequently, under moderate water deficit stress, flavonoid content increases in plants. For instance, studies have revealed that the most enriched drought-responsive genes are primarily involved in flavonoid biosynthesis and plant hormone signal transduction pathways. ABA-responsive elements binding factor (*ABF*) genes, *MYB* genes, *bHLH* genes, and flavonoid biosynthetic genes may form a regulatory network that collectively modulates drought-induced accumulation of flavonoid metabolites in blueberry leaves [90]. Among these, the ABA signaling pathway serves as the core of plant drought stress response, ABF transcription factors regulate the transcription of numerous ABRE-mediated downstream target genes that are significantly associated with flavonoid biosynthetic genes, including *4CL*, *C4H*, *CHS*, *DFR*, *F3H*, *F3′5′H*, *FLS*, *LAR*, *ANR*, *ANS*, and *UFGT* [90]. Research indicates that mild drought stress enhances the content of phenolics, anthocyanins, and other compounds in *Fragaria ananassa*, along with increased antioxidant activity [91]. These effects are accompanied by elevated levels of ABA and its derivatives, as well as upregulation of several genes involved in their biosynthesis, such as *PAL*, *C4H*, *4CL*, *DFR*, *ANS*, *FLS*, and *UFGT* [91]. In *A. thaliana*, the accumulation of anthocyanins and flavonols enhances reactive oxygen species (ROS) scavenging capacity and activates other stress responses, including osmotic adjustment and ion transport, thereby improving tolerance to drought stress [92].

Temperature regulates flavonoid biosynthesis in plants by influencing enzyme activity and gene expression. Numerous studies have demonstrated that low temperatures are more conducive to flavonoid accumulation (Figure 7). Appropriate low temperatures can induce anthocyanin biosynthesis in most plants, for instance, low temperature enhances the activity of enzymes in the flavonoid metabolic pathway in *Malus sieversii* f. *niedzwetzkyana*, thereby promoting flavonoid accumulation [74]. Research has revealed that *MdbHLH33* can directly bind to the low-temperature-responsive (LTR) cis-element in the *MdMYBPA1* promoter and activate its expression [74]. Furthermore, under low-temperature stress, the coordinated expression of *MdMYBPA1* and *MdbHLH33* in *Malus sieversii* f. *niedzwetzkyana* results in increased anthocyanin production in calli [74]. Within a certain range, anthocyanin content initially increases and then decreases with rising temperature, while high temperatures exhibit inhibitory effects. Elevated temperatures reduce anthocyanin synthesis rates while accelerating degradation rates. Studies indicate that high ambient temperatures suppress anthocyanin biosynthesis through the E3 ubiquitin ligase COP1 and the positive regulator of anthocyanin biosynthesis HY5 [93]. The bZIP transcription factor HY5, a positive regulator of anthocyanin biosynthesis, directly controls the expression of multiple early-stage and late-stage anthocyanin biosynthetic genes, research demonstrates that high temperatures primarily inhibit anthocyanin biosynthesis by destabilizing HY5 protein [93]. The stability of HY5 protein is known to be light-regulated through COP1-mediated degradation, indicating that COP1 participates in the high-temperature suppression of anthocyanins by promoting HY5 destabilization [93]. Additionally, HY5 can directly or indirectly negatively regulate *AtMYBL2* expression through miRNA858a activity [93]. As a negative regulator of anthocyanin biosynthesis, AtMYBL2 also mediates the high-temperature suppression of anthocyanin production [93].

Heavy metal stress imposes multiple detrimental effects on plants, including inhibited cell growth, chlorophyll degradation, impaired photosynthesis and respiration, nutrient consumption, lipid peroxidation, and membrane disintegration, ultimately leading to compromised crop growth or even mortality [94]. Plants can detoxify toxic metal(loid)s by accumulating various metabolites. Beyond scavenging excess reactive oxygen species (ROS) induced by metal(loid)s, certain metabolites can chelate metal(loid) ions [95]. The study found that anthocyanins can mitigate heavy metal toxicity in plants by scavenging free radicals, activating the antioxidant system, and chelating and sequestering heavy metals [94]. Nickel (Ni) is an essential element for plant growth and development, but excessive accumulation of Ni in soil can cause plant damage. Therefore, elucidating the molecular mechanisms underlying plant resistance to Ni stress is imperative. Studies have revealed that in *A. thaliana*, TT8 (AtbHLH42) acts as a positive regulator in response to Ni stress by upregulating six flavonoid biosynthetic enzyme-coding genes (*CHS*, *CHI*, *F3H*, *F3′H*, *DFR*, and *FLS*). This transcription factor participates in transcriptional regulation cascades during Ni stress response and promotes anthocyanin biosynthesis [96]. Cadmium (Cd), a predominant soil pollutant primarily derived from nonferrous metal smelting processes, exerts toxic effects on agricultural soils. Studies have identified that various flavonoid compounds and flavonoid metabolic genes are upregulated in plants under Cd stress [97]. Furthermore, genes associated with the metabolism of abscisic acid, brassinosteroid, ethylene, cytokinin, and jasmonate were all upregulated by Cd [97]. This suggests that flavonoid synthesis in Cd-stressed plants may be induced through endogenous hormones and signal transduction pathways.

Salinity stress represents one of the most prevalent abiotic stresses in nature. Soil salinization restricts plant growth and development, while high-salinity stress can induce severe oxidative damage in plants, triggering growth inhibition through cellular redox imbalance. Therefore, precise regulation of plant responses to salinity stress may improve crop performance in high-salinity environments [98,99]. Research has demonstrated that *AcCHS5*, regulated by AcMYB176, enhances salt tolerance in *Areca catechu* by modulating flavonoid biosynthesis and reactive oxygen species (ROS) scavenging. The transcription factor AcMYB176 directly activates *AcCHS5* by binding to its promoter region, functioning as a positive regulator to enhance flavonoid biosynthesis and scavenge reactive oxygen species (ROS), thereby improving salt tolerance in *Areca catechu* [99]. Additionally, studies in *A. thaliana* revealed that AtMYB111 acts as a positive regulator by interacting with *cis*-elements in the *AtCHS* promoter, promoting flavonoid accumulation under salinity stress [99].

Microplastics and nanoplastics represent one of the major global environmental challenges. Previous studies on the effects of microplastics and nanoplastics on plants primarily focused on their uptake processes or distribution within plant tissues. Recent research has demonstrated that flavonoid compounds play a crucial role in enhancing plant adaptability to nanoplastic stress [100]. Polystyrene nanoplastics (PSNPs) negatively affect seedling growth, primarily accumulating in plant roots and inhibiting growth through disruption of root microstructure and metabolic processes, thereby inducing phytotoxicity, oxidative stress, and nuclear damage [100]. Notably, studies revealed that PSNPs significantly upregulate the expression of most differentially expressed genes associated with flavonoid biosynthesis, promoting flavonoid accumulation and enhancing plant tolerance and detoxification capacity under PSNP stress [100]. Furthermore, investigations showed that *Arabidopsis*, poplar, and tomato all exhibited significantly increased total flavonoid levels in leaves following PSNP treatment, suggesting a conserved response to nanoplastic stress. Concurrently, earlier studies indicated that PSNPs can trigger defense responses in *Torreya grandis* by modulating the flavonoid biosynthetic pathway [100]. These findings collectively demonstrate that elevated flavonoid production helps mitigate intracellular ROS accumulation, activates downstream protective pathways, and reduces PSNP-induced damage, enabling resource reallocation to support normal growth and development [100].

## 7. Advances in Flavonoid Synthetic Biology

### 7.1. CRISPR/Cas9 Gene Editing Technology

With the advancement of synthetic biology, the application of gene editing technology to modify flavonoid biosynthetic pathways for the production of structurally and functionally specific flavonoid compounds has emerged as a research focus. CRISPR-associated protein 9 (CRISPR/Cas9) serves as a highly efficient genome editing tool in diverse plant species. CRISPR/Cas9-mediated gene editing represents a rapid, simple, efficient, and versatile technique for gene function analysis and crop improvement [101]. Previous studies have demonstrated that CRISPR/Cas9-mediated mutagenesis of the *F3H* gene can alter flower color in *Torenia fournieri*, resulting in pale blue (nearly white) flowers by modulating anthocyanin biosynthesis [102]. FtMYB45 functions as a negative regulator of flavonoid biosynthesis in Tartary buckwheat (*Fagopyrum tataricum*). Studies have demonstrated that CRISPR/Cas9-mediated targeted mutagenesis of *FtMYB45* via gene editing systems enhances the accumulation of multiple flavonoids in Tartary buckwheat [101]. Cinnamoyl-CoA reductase (CCR) catalyzes the reduction reaction for lignin biosynthesis, studies have shown that CRISPR/Cas9-mediated *in-planta* gene editing of the CCR gene enhances flavonoid biosynthesis in bamboo leaves while reducing lignin production [103]. Additionally, CRISPR/Cas9 knockout of the *DFR1*/*DFR2* genes in tobacco alters flower color by modulating flavonoid content, *DFR* knockout (*DFR*-KO) tobacco (*Nicotiana tabacum*) produces white flowers, whereas wild-type tobacco exhibits red flowers [104]. IFS is a key enzyme in isoflavonoid biosynthesis and shares the substrate naringenin with F3H and FNS II. By designing a CRISPR/Cas9 multiplex gene construct to simultaneously knockout *GmF3H1*, *GmF3H2*, and *GmFNSII-1* in soya bean (*Glycine max*), the competing branch pathways for IFS were blocked, thereby channeling more naringenin into the isoflavonoid metabolic pathway [105]. The results demonstrated a significant increase in isoflavonoid content in soya bean [105].

However, CRISPR/Cas9 gene-editing technology also has certain limitations. Off-target mutations represent a major constraint of CRISPR/Cas9 gene-editing technology. While this technology is designed to edit specific DNA sequences, the single-guide RNA (sgRNA) may occasionally affect other regions similar to the target sequence, leading to off-target mutations. These mutations can disrupt normal gene function and induce genomic instability [106]. The incidence of off-target mutations appears to exceed 50%, suggesting that overcoming this limitation could represent a significant advancement for CRISPR/Cas9 gene-editing technology [106].

### 7.2. Transgenic Engineering

Furthermore, genetic modification of flavonoid secondary metabolic pathways to produce desirable natural products represents an attractive approach in plant biotechnology [107]. Isoflavonoids, a class of plant flavonoid metabolites found exclusively in legumes, serve as key compounds mediating diverse plant-microbial interactions, therefore, metabolic engineering of isoflavonoids in more widely consumed non-legume vegetables, grains, and fruits to enhance dietary intake of these compounds holds significant research potential [107]. Through genetic engineering, soybean *IFS* was introduced into non-legume plants, including tobacco (*Nicotiana tabacum*), lettuce (*Lactuca sativa*), and petunia (*Petunia hybrida*), by employing a genetically modified secondary metabolism pathway, soybean *IFS* was successfully produced in these non-legume species [107]. The study demonstrated that overexpression of soybean *IFS* in tobacco, petunia, and lettuce led to the accumulation of isoflavonoids in the transgenic plants [107].

### 7.3. Microbial Synthesis

In recent years, traditional sources of flavonoids have become insufficient to meet current demands. With the elucidation of flavonoid biosynthetic pathways and advances in synthetic biology, the production of flavonoids using synthetic metabolic engineering approaches with microorganisms as hosts has become feasible. This includes both microbial mono-culture and microbial co-culture systems for flavonoid synthesis [108]. In microbial mono-culture systems for flavonoid production, *Escherichia coli* and *Saccharomyces cerevisiae* are commonly selected as microbial hosts [108]. Examples include the de novo synthesis of anthocyanins in *S. cerevisiae* [109], and the direct biosynthesis of naringenin from glucose in *E. coli* for efficient flavonoid production [110]. Recent studies have demonstrated that engineered *Lactococcus lactis* strains can generate anthocyanins from green tea [111]. Microbial co-culture for flavonoid synthesis involves cultivating two or more microbial strains to complete target biosynthetic pathways. This approach first modularizes the complete biosynthetic pathway, with each microorganism responsible for a specific module, compared with traditional mono-culture methods, microbial co-culture significantly reduces the biosynthetic burden on individual strains, lowers associated metabolic stress, and provides better resistance to environmental disturbances [108]. For instance, co-culture of *IFS* gene-carrying *S. cerevisiae* with (S)-naringenin-high-producing *E. coli* can generate isoflavonoids with a yield 17.6 times higher than *S. cerevisiae* mono-culture [112]. Beyond synthesizing free aglycones through co-culture strategies, various glycosylated flavonoids have also been successfully produced using microbial consortia, the microbial co-culture strategy provides new directions for improving flavonoid production efficiency and controlling manufacturing costs [108].

However, plants and microorganisms are two entirely distinct systems, exhibiting significant differences in cell structure and subcellular organelles, the architecture and composition of cell walls also differ, leading to variations in flavonoid biosynthesis. For instance, the plant secondary metabolite anthocyanin can be stored in vacuoles, whereas natural products obtained through engineered microorganisms (such as (*2S*)-naringenin) may be partially secreted extracellularly or retained intracellularly [113]. In engineered microorganisms, the production of natural products is achieved through non-natural selection, resulting in uncertainties regarding the mechanisms of natural product synthesis and subcellular organelle transport [113].

Furthermore, the metabolic engineering of flavonoid production using microorganisms as hosts faces numerous technical challenges. For instance, enzymes involved in flavonoid biosynthesis generally exhibit low activity in microbial cell factories. Naturally occurring high-activity flavonoid synthases screened through conventional methods are often insufficiently active to meet the demands of target compound synthesis. Studies have shown that directed evolution can enhance enzyme performance, but the screening workload remains a bottleneck [113]. Flavonoid-deriving enzymes display promiscuity, enabling them to participate in both plant-specific metabolic reactions and other reactions [113]. However, enzyme promiscuity can lead to cross-talk between different metabolic pathways and reduced specificity, potentially decreasing substrate catalytic efficiency. Although various methods have been developed to optimize the heterologous synthesis of flavonoids in cell factories, and numerous engineered strains have been obtained, predicting the optimal expression combination remains challenging [113]. Traditional analytical methods suffer from low detection efficiency and time-consuming procedures, making it difficult to screen high-titer strains. The lack of high-throughput screening methods has hindered research on key enzymes and high-yield flavonoid-producing engineered strains. A prerequisite for efficient flavonoid production by engineered microorganisms is maintaining robust cellular vitality. However, stresses such as product toxicity and metabolic imbalance can negatively impact cell viability, interfere with metabolic processes, and damage cellular structures like cell membranes [113]. Therefore, improving the tolerance of engineered strains to flavonoid compounds will be a crucial research direction. Additionally, the low yields of heterologous flavonoid synthesis in engineered microorganisms remain a major limiting factor for large-scale industrial production and commercial applications [113].

## 8. Prospects

Flavonoids are highly bioactive secondary metabolites in plants that play crucial roles in stress resistance. With advances in molecular biology techniques, the biosynthetic pathways and regulatory mechanisms of plant flavonoids have been gradually elucidated. This review provides a comprehensive summary of the biosynthetic processes and regulation of plant flavonoids, offering a theoretical foundation for further development and utilization of these compounds. However, many unknowns remain to be explored. For instance, although we have gained some understanding of the important regulatory roles of MYB family proteins, particularly R2R3-MYBs, in flavonoid biosynthesis, the functions of many MYB transcription factors remain uncharacterized. Future studies should focus on elucidating the roles of MYB transcription factors and their specific functions in different plant species and tissues. Additionally, the interactions among various regulators and enzymes in the flavonoid biosynthetic pathway, as well as the precise mechanisms by which they respond to environmental signals, require further investigation. Another critical research direction involves applying these findings to effectively enhance flavonoid content and bioactivity in crops, addressing the growing demand for health-promoting foods and natural pharmaceuticals.

In the future, with a deeper understanding of the flavonoid biosynthetic pathway and its regulatory mechanisms, it will be possible to more precisely design strategies such as gene editing, genetic modification, and genetic engineering to optimize flavonoid production. Additionally, by integrating high-throughput sequencing technologies and bioinformatics analysis, novel targets and strategies can be identified for the genetic improvement and molecular breeding of flavonoids. Collectively, these studies will not only enhance our understanding of flavonoid biosynthesis and regulation but also provide new opportunities for agricultural production and medical applications.

## Figures and Tables

**Table 1:** Key enzymes involved in the synthesis of plant flavonoids.

No.	Enzyme	Abbreviation	EC Number	Gene Numbering in *A. thaliana*
1	Phenylalanine ammonia-lyase	PAL	4.3.1.24	AT2G37040 (*PAL1*) AT3G53260 (*PAL2*) AT5G04230 (*PAL3*) AT3G10340 (*PAL4*)
2	Cinnamic acid 4-hydroxylase	C4H	1.14.14.91	AT2G30490 (*C4H*)
3	4-Coumarate-CoA ligase	4CL	6.2.1.12	AT1G51680 (*4CL1*) AT3G21240 (*4CL2*) AT1G65060 (*4CL3*) AT3G21230 (*4CL5*) AT1G62940 (*ACOS5*) AT4G05160 AT4G19010 AT5G63380
4	Chalcone synthase	CHS	2.3.1.74	AT5G13930 (*TT4*)
5	Chalcone reductase	CHR	None	None
6	Chalcone isomerase	CHI	5.5.1.6	AT3G55120 (*TT5*) AT5G05270 (*CHIL*) AT5G66220
7	Isoflavonoid synthase	IFS	1.14.14.87	None
8	Flavone synthase	FNS	1.14.20.5 1.14.19.76	None
9	Flavanone 3-hydroxylase	F3H	1.14.11.9	AT3G51240 (*F3H*)
10	Flavonol synthase	FLS	1.14.20.6	AT5G08640 (*FLS1*) AT5G63580 (*FLS2*) AT5G63590 (*FLS3*) AT5G63595 (*FLS4*) AT5G63600 (*FLS5*) AT5G43935 (*FLS6*)
11	Flavonoid 3′,5′-hydroxylase	F3′5′H	1.14.14.81	None
12	Flavonoid 3′-hydroxylase	F3′H	1.14.14.82	AT5G07990 (*TT7*)
13	Dihydroflavonol 4-reductase	DFR	1.1.1.219	AT5G42800 (*DFR*)
14	Anthocyanidin synthase	ANS	1.14.20.4	AT4G22870 AT4G22880 (*LDOX*)
15	Leucoanthocyanidin reductase	LAR	1.17.1.3	None
16	Anthocyanidin reductase	ANR	1.3.1.77	AT1G61720 (*BAN*)

**Table 2:** Information of AtMYB transcription factors that regulate flavonoid synthesis.

Gene Name	Gene ID	Subgroup	Promote or Inhibit
*AtMYB21*	AT3G27810	R2R3subgroup19	Promote
*AtMYB24*	AT5G40350	R2R3subgroup19	Promote
*AtMYB57*	AT3G01530	R2R3	Promote
*AtMYB99/ATMYBCU15*	AT5G62320	R2R3	Promote
*AtMYB11/PFG2*	AT3G62610	R2R3subgroup7	Promote
*AtMYB12/PFG1*	AT2G47460	R2R3subgroup7	Promote
*AtMYB111/PFG3*	AT5G49330	R2R3subgroup7	Promote
*AtMYB123/TT2*	AT5G35550	R2R3subgroup5	Promote
*AtMYB5*	AT3G13540	R2R3	Promote
*AtMYB114*	AT1G66380	R2R3	Promote
*AtMYB113*	AT1G66370	R2R3subgroup6	Promote
*AtMYB75/PAP1*	AT1G56650	R2R3subgroup6	Promote
*AtMYB90/PAP2*	AT1G66390	R2R3subgroup6	Promote
*AtMYB112*	AT1G48000	R2R3subgroup20	Promote/Inhibit
*AtMYB7/ATY49*	AT2G16720	R2R3subgroup4	Inhibit
*AtMYB4*	AT4G38620	R2R3subgroup4	Inhibit
*AtMYB32*	AT4G34990	R2R3subgroup4	Inhibit
*AtMYB3*	AT1G22640	R2R3subgroup4	Inhibit
*AtMYB6*	AT4G09460	R2R3	Inhibit
*AtMYBL2*	AT1G71030	R3	Inhibit

## Data Availability

All the data used in this review paper are available online.

## References

[1] Stafford H.A. (1991). Flavonoid Evolution: An Enzymic Approach. Plant Physiol..

[2] Yonekura-Sakakibara K., Higashi Y., Nakabayashi R. (2019). The Origin and Evolution of Plant Flavonoid Metabolism. Front. Plant Sci..

[3] Wu J., Lv S., Zhao L., Gao T., Yu C., Hu J., Ma F. (2023). Advances in the study of the function and mechanism of the action of flavonoids in plants under environmental stresses. Planta.

[4] Davies K.M., Andre C.M., Kulshrestha S., Zhou Y., Schwinn K.E., Albert N.W., Chagné D., van Klink J.W., Landi M., Bowman J.L. (2024). The evolution of flavonoid biosynthesis. Philos. Trans. R. Soc. B Biol. Sci..

[5] Panche A.N., Diwan A.D., Chandra S.R. (2016). Flavonoids: An overview. J. Nutr. Sci..

[6] Procházková D., Boušová I., Wilhelmová N. (2011). Antioxidant and prooxidant properties of flavonoids. Fitoterapia.

[7] Hu L., Luo Y., Yang J., Cheng C. (2025). Botanical Flavonoids: Efficacy, Absorption, Metabolism and Advanced Pharmaceutical Technology for Improving Bioavailability. Molecules.

[8] Sun C., Zhang M., Dong H., Liu W., Guo L., Wang X. (2020). A spatially-resolved approach to visualize the distribution and biosynthesis of flavones in Scutellaria baicalensis Georgi. J. Pharm. Biomed. Anal..

[9] Liu W., Feng Y., Yu S., Fan Z., Li X., Li J., Yin H. (2021). The Flavonoid Biosynthesis Network in Plants. Int. J. Mol. Sci..

[10] Wang H., Liu S., Wang T., Liu H., Xu X., Chen K., Zhang P. (2020). The moss flavone synthase I positively regulates the tolerance of plants to drought stress and UV-B radiation. Plant Sci..

[11] Jaakola L., Hohtola A. (2010). Effect of latitude on flavonoid biosynthesis in plants. Plant Cell Environ..

[12] Teale W.D., Pasternak T., Bosco C.D., Dovzhenko A., Kratzat K., Bildl W., Schwörer M., Falk T., Ruperti B., Schaefer J.V. (2020). Flavonol-mediated stabilization of PIN efflux complexes regulates polar auxin transport. EMBO J..

[13] Tan J., Han Y., Han B., Qi X., Cai X., Ge S., Xue H. (2022). Extraction and purification of anthocyanins: A review. J. Agric. Food Res..

[14] Teles Y.C.F., Souza M.S.R., Souza M.D.F.V.d. (2018). Sulphated Flavonoids: Biosynthesis, Structures, and Biological Activities. Molecules.

[15] Liu A., Zhu Y., Wang Y., Wang T., Zhao S., Feng K., Li L., Wu P. (2023). Molecular identification of phenylalanine ammonia lyase-encoding genes EfPALs and EfPAL2-interacting transcription factors in Euryale ferox. Front. Plant Sci..

[16] Ferreyra M.L.F., Rius S.P., Casati P. (2012). Flavonoids: Biosynthesis, biological functions, and biotechnological applications. Front. Plant Sci..

[17] Liu T., Liu T., Zhang X., Song J., Qiu Y., Yang W., Jia H., Wang H., Li X. (2023). Combined widely targeted metabolomics and transcriptomics analysis reveals differentially accumulated metabolites and the underlying molecular bases in fleshy taproots of distinct radish genotypes. Plant Physiol. Biochem..

[18] Lapcik O., Honys D., Koblovska R., Mackova Z., Vitkova M., Klejdus B. (2006). Isoflavonoids are present in Arabidopsis thaliana despite the absence of any homologue to known isoflavonoid synthases. Plant Physiol. Biochem..

[19] Xie C., Zhan T., Huang J., Lan J., Shen L., Wang H., Zheng X. (2023). Functional characterization of nine critical genes encoding rate-limiting enzymes in the flavonoid biosynthesis of the medicinal herb Grona styracifolia. BMC Plant Biol..

[20] Leonard E., Yan Y., Lim K.H., Koffas M.A.G. (2005). Investigation of Two Distinct Flavone Synthases for Plant-Specific Flavone Biosynthesis inSaccharomyces cerevisiae. Appl. Environ. Microbiol..

[21] Feng Y., Tian X., Liang W., Nan X., Zhang A., Li W., Ma Z. (2023). Genome-wide identification of grape ANS gene family and expression analysis at different fruit coloration stages. BMC Plant Biol..

[22] Cao Y., Li K., Li Y., Zhao X., Wang L. (2020). MYB Transcription Factors as Regulators of Secondary Metabolism in Plants. Biology.

[23] Wu Y., Wen J., Xia Y., Zhang L., Du H. (2022). Evolution and functional diversification of R2R3-MYB transcription factors in plants. Hortic. Res..

[24] Stracke R., Werber M., Weisshaar B. (2001). The R2R3-MYB gene family in Arabidopsis thaliana. Curr. Opin. Plant Biol..

[25] Chezem W.R., Memon A., Li F.-S., Weng J.-K., Clay N.K. (2017). SG2-Type R2R3-MYB Transcription Factor MYB15 Controls Defense-Induced Lignification and Basal Immunity in Arabidopsis. Plant Cell.

[26] Lotkowska M.E., Tohge T., Fernie A.R., Xue G.-P., Balazadeh S., Mueller-Roeber B. (2015). The Arabidopsis transcription factor MYB112 promotes anthocyanin formation during salinity and under high light stress. Plant Physiol..

[27] Shi M.-Z., Xie D.-Y. (2014). Biosynthesis and Metabolic Engineering of Anthocyanins in Arabidopsis thaliana. Recent Pat. Biotechnol..

[28] Stracke R., Ishihara H., Huep G., Barsch A., Mehrtens F., Niehaus K., Weisshaar B. (2007). Differential regulation of closely related R2R3-MYB transcription factors controls flavonol accumulation in different parts of the Arabidopsis thaliana seedling. Plant J..

[29] Zhang X., He Y., Li L., Liu H., Hong G., Hancock R. (2021). Involvement of the R2R3-MYB transcription factor MYB21 and its homologs in regulating flavonol accumulation in Arabidopsis stamen. J. Exp. Bot..

[30] Zhong R., Ye Z.-H. (2012). MYB46 and MYB83 Bind to the SMRE Sites and Directly Activate a Suite of Transcription Factors and Secondary Wall Biosynthetic Genes. Plant Cell Physiol..

[31] Zimmermann I.M., Heim M.A., Weisshaar B., Uhrig J.F. (2004). Comprehensive identification of Arabidopsis thaliana MYB transcription factors interacting with R/B-like BHLH proteins. Plant J..

[32] Battat M., Eitan A., Rogachev I., Hanhineva K., Fernie A., Tohge T., Beekwilder J., Aharoni A. (2019). A MYB Triad Controls Primary and Phenylpropanoid Metabolites for Pollen Coat Patterning. Plant Physiol..

[33] Gonzalez A., Mendenhall J., Huo Y., Lloyd A. (2009). TTG1 complex MYBs, MYB5 and TT2, control outer seed coat differentiation. Dev. Biol..

[34] Rowan D.D., Cao M., Lin-Wang K., Cooney J.M., Jensen D.J., Austin P.T., Hunt M.B., Norling C., Hellens R.P., Schaffer R.J. (2009). Environmental regulation of leaf colour in red 35S:PAP1 Arabidopsis thaliana. New Phytol..

[35] Wang B., Luo Q., Li Y., Yin L., Zhou N., Li X., Gan J., Dong A. (2020). Structural insights into target DNA recognition by R2R3-MYB transcription factors. Nucleic Acids Res..

[36] Prouse M.B., Campbell M.M., Unver T. (2013). Interactions between the R2R3-MYB transcription factor, AtMYB61, and target DNA binding sites. PLoS ONE.

[37] Zhou M., Wei L., Sun Z., Gao L., Meng Y., Tang Y., Wu Y. (2015). Production and transcriptional regulation of proanthocyanidin biosynthesis in forage legumes. Appl. Microbiol. Biotechnol..

[38] Solfanelli C., Poggi A., Loreti E., Alpi A., Perata P. (2006). Sucrose-Specific Induction of the Anthocyanin Biosynthetic Pathway in Arabidopsis. Plant Physiol..

[39] Jeong C.Y., Kim J.H., Lee W.J., Jin J.Y., Kim J., Hong S.-W., Lee H. (2018). AtMyb56 Regulates Anthocyanin Levels via the Modulation of AtGPT2 Expression in Response to Sucrose in Arabidopsis. Mol. Cells.

[40] Jin H., Cominelli E., Bailey P., Parr A., Mehrtens F., Jones J., Tonelli C., Weisshaar B., Martin C. (2000). Transcriptional repression by AtMYB4 controls production of UV-protecting sunscreens in Arabidopsis. EMBO J..

[41] Fornalé S., Lopez E., Salazar-Henao J.E., Fernández-Nohales P., Rigau J., Caparros-Ruiz D. (2014). AtMYB7, a New Player in the Regulation of UV-Sunscreens in Arabidopsis thaliana. Plant Cell Physiol..

[42] Preston J., Wheeler J., Heazlewood J., Li S.F., Parish R.W. (2004). AtMYB32 is required for normal pollen development in Arabidopsis thaliana. Plant J..

[43] Dubos C., Le Gourrierec J., Baudry A., Huep G., Lanet E., Debeaujon I., Routaboul J.-M., Alboresi A., Weisshaar B., Lepiniec L. (2008). MYBL2 is a new regulator of flavonoid biosynthesis in Arabidopsis thaliana. Plant J..

[44] Matsui K., Umemura Y., Ohme-Takagi M. (2008). AtMYBL2, a protein with a single MYB domain, acts as a negative regulator of anthocyanin biosynthesis in Arabidopsis. Plant J..

[45] Li S. (2014). Transcriptional control of flavonoid biosynthesis. Plant Signal. Behav..

[46] Viola I.L., Camoirano A., Gonzalez D.H. (2016). Redox-Dependent Modulation of Anthocyanin Biosynthesis by the TCP Transcription Factor TCP15 during Exposure to High Light Intensity Conditions in Arabidopsis. Plant Physiol..

[47] Li S., Zachgo S. (2013). TCP3 interacts with R2R3-MYB proteins, promotes flavonoid biosynthesis and negatively regulates the auxin response in Arabidopsis thaliana. Plant J..

[48] Gou J.-Y., Felippes F.F., Liu C.-J., Weigel D., Wang J.-W. (2011). Negative regulation of anthocyanin biosynthesis in Arabidopsis by a miR156-targeted SPL transcription factor. Plant Cell.

[49] Morishita T., Kojima Y., Maruta T., Nishizawa-Yokoi A., Yabuta Y., Shigeoka S. (2009). Arabidopsis NAC Transcription Factor, ANAC078, Regulates Flavonoid Biosynthesis under High-light. Plant Cell Physiol..

[50] Li S. (2015). The Arabidopsis thaliana TCP transcription factors: A broadening horizon beyond development. Plant Signal Behav..

[51] Zhao X., Wu T., Guo S., Hu J., Zhan Y. (2022). Ectopic Expression of AeNAC83, a NAC Transcription Factor from Abelmoschus esculentus, Inhibits Growth and Confers Tolerance to Salt Stress in Arabidopsis. Int. J. Mol. Sci..

[52] Iwakawa H.-o., Tomari Y. (2015). The Functions of MicroRNAs: mRNA Decay and Translational Repression. Trends Cell Biol..

[53] Bonar N., Liney M., Zhang R., Austin C., Dessoly J., Davidson D., Stephens J., McDougall G., Taylor M., Bryan G.J. (2018). Potato miR828 Is Associated With Purple Tuber Skin and Flesh Color. Front. Plant Sci..

[54] Luo Q.-J., Mittal A., Jia F., Rock C.D. (2011). An autoregulatory feedback loop involving PAP1 and TAS4 in response to sugars in Arabidopsis. Plant Mol. Biol..

[55] Wu G., Cao A., Wen Y., Bao W., She F., Wu W., Zheng S., Yang N. (2023). Characteristics and Functions of MYB (v-Myb avivan myoblastsis virus oncogene homolog)-Related Genes in Arabidopsis thaliana. Genes.

[56] Loreti E., Povero G., Novi G., Solfanelli C., Alpi A., Perata P. (2008). Gibberellins, jasmonate and abscisic acid modulate the sucrose-induced expression of anthocyanin biosynthetic genes in Arabidopsis. New Phytol..

[57] Song R.-F., Hu X.-Y., Liu W.-C., Yuan H.-M. (2024). ABA functions in low phosphate-induced anthocyanin accumulation through the transcription factor ABI5 in Arabidopsis. Plant Cell Rep..

[58] Song S., Liu B., Song J., Pang S., Song T., Gao S., Zhang Y., Huang H., Qi T. (2022). A molecular framework for signaling crosstalk between jasmonate and ethylene in anthocyanin biosynthesis, trichome development, and defenses against insect herbivores in Arabidopsis. J. Integr. Plant Biol..

[59] Lee S.-H., Kim S.-H., Park T.-K., Kim Y.-P., Lee J.-W., Kim T.-W. (2024). Transcription factors BZR1 and PAP1 cooperate to promote anthocyanin biosynthesis in Arabidopsis shoots. Plant Cell.

[60] Bühler E., Fahrbach E., Schaller A., Stührwohldt N. (2023). Sulfopeptide CLEL6 inhibits anthocyanin biosynthesis in Arabidopsis thaliana. Plant Physiol..

[61] Cheng C., Guo Z., Li H., Mu X., Wang P., Zhang S., Yang T., Cai H., Wang Q., Lü P. (2022). Integrated metabolic, transcriptomic and chromatin accessibility analyses provide novel insights into the competition for anthocyanins and flavonols biosynthesis during fruit ripening in red apple. Front. Plant Sci..

[62] Zhong C., Tang Y., Pang B., Li X., Yang Y., Deng J., Feng C., Li L., Ren G., Wang Y. (2020). The R2R3-MYB transcription factor GhMYB1a regulates flavonol and anthocyanin accumulation in Gerbera hybrida. Hortic. Res..

[63] Liu L., Zhang L.-Y., Wang S.-L., Niu X.-Y. (2016). Analysis of anthocyanins and flavonols in petals of 10 Rhododendron species from the Sygera Mountains in Southeast Tibet. Plant Physiol. Biochem..

[64] Chen W., Xiao Z., Wang Y., Wang J., Zhai R., Lin-Wang K., Espley R., Ma F., Li P. (2021). Competition between anthocyanin and kaempferol glycosides biosynthesis affects pollen tube growth and seed set of Malus. Hortic. Res..

[65] Yuan Y.-W., Rebocho A.B., Sagawa J.M., Stanley L.E., Bradshaw H.D. (2016). Competition between anthocyanin and flavonol biosynthesis produces spatial pattern variation of floral pigments between Mimulus species. Proc. Natl. Acad. Sci. USA.

[66] Maloney G.S., DiNapoli K.T., Muday G.K. (2014). The anthocyanin reduced Tomato Mutant Demonstrates the Role of Flavonols in Tomato Lateral Root and Root Hair Development. Plant Physiol..

[67] Luo P., Ning G., Wang Z., Shen Y., Jin H., Li P., Huang S., Zhao J., Bao M. (2016). Disequilibrium of Flavonol Synthase and Dihydroflavonol-4-Reductase Expression Associated Tightly to White vs. Red Color Flower Formation in Plants. Front. Plant Sci..

[68] Yang J., Wu X., Aucapiña C.b., Zhang D., Huang J., Hao Z., Zhang Y., Ren Y., Miao Y. (2023). NtMYB12 requires for competition between flavonol and (pro)anthocyanin biosynthesis in Narcissus tazetta tepals. Mol. Hortic..

[69] Anwar M., Yu W., Yao H., Zhou P., Allan A.C., Zeng L. (2019). NtMYB3, an R2R3-MYB from Narcissus, Regulates Flavonoid Biosynthesis. Int. J. Mol. Sci..

[70] Nakatsuka T., Saito M., Yamada E., Fujita K., Kakizaki Y., Nishihara M. (2012). Isolation and characterization of GtMYBP3 and GtMYBP4, orthologues of R2R3-MYB transcription factors that regulate early flavonoid biosynthesis, in gentian flowers. J. Exp. Bot..

[71] Xu T., Yu L., Huang N., Liu W., Fang Y., Chen C., Jiang L., Wang T., Zhao J., Zhang Z. (2023). The regulatory role of MdNAC14-Like in anthocyanin synthesis and proanthocyanidin accumulation in red-fleshed apples. Plant Physiol. Biochem..

[72] Cheng J., Yu K., Shi Y., Wang J., Duan C. (2021). Transcription Factor VviMYB86 Oppositely Regulates Proanthocyanidin and Anthocyanin Biosynthesis in Grape Berries. Front. Plant Sci..

[73] Vale M., Rodrigues J., Badim H., Gerós H., Conde A. (2021). Exogenous Application of Non-mature miRNA-Encoded miPEP164c Inhibits Proanthocyanidin Synthesis and Stimulates Anthocyanin Accumulation in Grape Berry Cells. Front. Plant Sci..

[74] Wang N., Qu C., Jiang S., Chen Z., Xu H., Fang H., Su M., Zhang J., Wang Y., Liu W. (2018). The proanthocyanidin-specific transcription factor MdMYBPA1 initiates anthocyanin synthesis under low-temperature conditions in red-fleshed apples. Plant J..

[75] Liu C., Jun J.H., Dixon R.A. (2014). MYB5 and MYB14 Play Pivotal Roles in Seed Coat Polymer Biosynthesis in Medicago truncatula. Plant Physiol..

[76] Jiang X., Huang K., Zheng G., Hou H., Wang P., Jiang H., Zhao X., Li M., Zhang S., Liu Y. (2018). CsMYB5a and CsMYB5e from *Camellia sinensis* differentially regulate anthocyanin and proanthocyanidin biosynthesis. Plant Sci..

[77] Shamsudin N.F., Ahmed Q.U., Mahmood S., Shah S.A.A., Khatib A., Mukhtar S., Alsharif M.A., Parveen H., Zakaria Z.A. (2022). Antibacterial Effects of Flavonoids and Their Structure-Activity Relationship Study: A Comparative Interpretation. Molecules.

[78] Dwivedi K., Mandal A.K., Afzal O., Altamimi A.S.A., Sahoo A., Alossaimi M.A., Almalki W.H., Alzahrani A., Barkat A., Almeleebia T.M. (2023). Emergence of Nano-Based Formulations for Effective Delivery of Flavonoids against Topical Infectious Disorders. Gels.

[79] Chen Y.-Y., Lu H.-Q., Jiang K.-X., Wang Y.-R., Wang Y.-P., Jiang J.-J. (2022). The Flavonoid Biosynthesis and Regulation in Brassica napus: A Review. Int. J. Mol. Sci..

[80] Ullah C., Tsai C.-J., Unsicker S.B., Xue L., Reichelt M., Gershenzon J., Hammerbacher A. (2018). Salicylic acid activates poplar defense against the biotrophic rust fungusMelampsora larici-populinavia increased biosynthesis of catechin and proanthocyanidins. New Phytol..

[81] Montesinos Á., Sacristán S., del Prado-Polonio P., Arnaiz A., Díaz-González S., Diaz I., Santamaria M.E. (2024). Contrasting plant transcriptome responses between a pierce-sucking and a chewing herbivore go beyond the infestation site. BMC Plant Biol..

[82] Chin S., Behm C.A., Mathesius U. (2018). Functions of Flavonoids in Plant–Nematode Interactions. Plants.

[83] Schenke D., BÖTtcher C., Scheel D. (2011). Crosstalk between abiotic ultraviolet-B stress and biotic (flg22) stress signalling in Arabidopsis prevents flavonol accumulation in favor of pathogen defence compound production. Plant Cell Environ..

[84] Ye J.-H., Lv Y.-Q., Liu S.-R., Jin J., Wang Y.-F., Wei C.-L., Zhao S.-Q. (2021). Effects of Light Intensity and Spectral Composition on the Transcriptome Profiles of Leaves in Shade Grown Tea Plants (*Camellia sinensis* L.) and Regulatory Network of Flavonoid Biosynthesis. Molecules.

[85] Liu L., Li Y., She G., Zhang X., Jordan B., Chen Q., Zhao J., Wan X. (2018). Metabolite profiling and transcriptomic analyses reveal an essential role of UVR8-mediated signal transduction pathway in regulating flavonoid biosynthesis in tea plants (*Camellia sinensis*) in response to shading. BMC Plant Biol..

[86] Zheng C., Ma J.-Q., Ma C.-L., Shen S.-Y., Liu Y.-F., Chen L. (2019). Regulation of Growth and Flavonoid Formation of Tea Plants (*Camellia sinensis*) by Blue and Green Light. J. Agric. Food Chem..

[87] Wang P., Chen S., Gu M., Chen X., Chen X., Yang J., Zhao F., Ye N. (2020). Exploration of the Effects of Different Blue LED Light Intensities on Flavonoid and Lipid Metabolism in Tea Plants via Transcriptomics and Metabolomics. Int. J. Mol. Sci..

[88] Zoratti L., Karppinen K., Escobar A.L., HÃ¤ggman H., Jaakola L. (2014). Light-controlled flavonoid biosynthesis in fruits. Front. Plant Sci..

[89] Liu Y.-Y., Chen X.-R., Wang J.-P., Cui W.-Q., Xing X.-X., Chen X.-Y., Ding W.-Y., God’spower B.-O., Eliphaz N., Sun M.-Q. (2019). Transcriptomic analysis reveals flavonoid biosynthesis of Syringa oblata Lindl. in response to different light intensity. BMC Plant Biol..

[90] Feng X., Bai S., Zhou L., Song Y., Jia S., Guo Q., Zhang C. (2024). Integrated Analysis of Transcriptome and Metabolome Provides Insights into Flavonoid Biosynthesis of Blueberry Leaves in Response to Drought Stress. Int. J. Mol. Sci..

[91] Perin E.C., Messias R.d.S., Borowski J.M., Crizel R.L., Schott I.B., Carvalho I.R., Rombaldi C.V., Galli V. (2019). ABA-dependent salt and drought stress improve strawberry fruit quality. Food Chem..

[92] Nakabayashi R., Yonekura-Sakakibara K., Urano K., Suzuki M., Yamada Y., Nishizawa T., Matsuda F., Kojima M., Sakakibara H., Shinozaki K. (2013). Enhancement of oxidative and drought tolerance in Arabidopsis by overaccumulation of antioxidant flavonoids. Plant J..

[93] Kim S., Hwang G., Lee S., Zhu J.-Y., Paik I., Nguyen T.T., Kim J., Oh E. (2017). High Ambient Temperature Represses Anthocyanin Biosynthesis through Degradation of HY5. Front. Plant Sci..

[94] Li Z., Ahammed G.J. (2023). Hormonal regulation of anthocyanin biosynthesis for improved stress tolerance in plants. Plant Physiol. Biochem..

[95] Ahammed G.J., Yang Y. (2022). Anthocyanin-mediated arsenic tolerance in plants. Environ. Pollut..

[96] Hu Y., Peng Y., Qi X. (2025). The TT8 transcription factor alleviates nickel toxicity in Arabidopsis. Biochem. Biophys. Res. Commun..

[97] Wang Y., Cui T., Niu K., Ma H. (2024). Co-expression analyses reveal key Cd stress response-related metabolites and transcriptional regulators in Kentucky bluegrass. Chemosphere.

[98] Kim D., Jeon S.J., Yanders S., Park S.-C., Kim H.S., Kim S. (2022). MYB3 plays an important role in lignin and anthocyanin biosynthesis under salt stress condition in Arabidopsis. Plant Cell Rep..

[99] Jiang Y., Khan N.M., Ali A., Zhou G., Zhou Y., Li P., Wan Y. (2025). AcMYB176-Regulated AcCHS5 Enhances Salt Tolerance in Areca catechu by Modulating Flavonoid Biosynthesis and Reactive Oxygen Species Scavenging. Int. J. Mol. Sci..

[100] Cui J., Li X., Gan Q., Lu Z., Du Y., Noor I., Wang L., Liu S., Jin B. (2024). Flavonoids Mitigate Nanoplastic Stress in Ginkgo biloba. Plant Cell Environ..

[101] Wen D., Wu L., Wang M., Yang W., Wang X., Ma W., Sun W., Chen S., Xiang L., Shi Y. (2022). CRISPR/Cas9-Mediated Targeted Mutagenesis of FtMYB45 Promotes Flavonoid Biosynthesis in Tartary Buckwheat (*Fagopyrum tataricum*). Front. Plant Sci..

[102] Nishihara M., Higuchi A., Watanabe A., Tasaki K. (2018). Application of the CRISPR/Cas9 system for modification of flower color in Torenia fournieri. BMC Plant Biol..

[103] Sun H., Wang S., Zhu C., Yang K., Liu Y., Gao Z. (2023). A new biotechnology for in-planta gene editing and its application in promoting flavonoid biosynthesis in bamboo leaves. Plant Methods.

[104] Jiang J., Huang H., Gao Q., Li Y., Xiang H., Zeng W., Xu L., Liu X., Li J., Mi Q. (2023). Effects of editing DFR genes on flowers, leaves, and roots of tobacco. BMC Plant Biol..

[105] Zhang P., Du H., Wang J., Pu Y., Yang C., Yan R., Yang H., Cheng H., Yu D. (2019). Multiplex CRISPR/Cas9-mediated metabolic engineering increases soya bean isoflavone content and resistance to soya bean mosaic virus. Plant Biotechnol. J..

[106] Vaghari-Tabari M., Hassanpour P., Sadeghsoltani F., Malakoti F., Alemi F., Qujeq D., Asemi Z., Yousefi B. (2022). CRISPR/Cas9 gene editing: A new approach for overcoming drug resistance in cancer. Cell. Mol. Biol. Lett..

[107] Liu R., Hu Y., Li J., Lin Z. (2007). Production of soybean isoflavone genistein in non-legume plants via genetically modified secondary metabolism pathway. Metab. Eng..

[108] Lou H., Hu L., Lu H., Wei T., Chen Q. (2021). Metabolic Engineering of Microbial Cell Factories for Biosynthesis of Flavonoids: A Review. Molecules.

[109] Eichenberger M., Hansson A., Fischer D., Dürr L., Naesby M. (2018). De novo biosynthesis of anthocyanins in Saccharomyces cerevisiae. FEMS Yeast Res..

[110] Zhou S., Hao T., Zhou J. (2020). Fermentation and Metabolic Pathway Optimization to De Novo Synthesize (2S)-Naringenin in Escherichia coli. J. Microbiol. Biotechnol..

[111] Solopova A., van Tilburg A.Y., Foito A., Allwood J.W., Stewart D., Kulakauskas S., Kuipers O.P. (2019). Engineering Lactococcus lactis for the production of unusual anthocyanins using tea as substrate. Metab. Eng..

[112] Katsuyama Y., Miyahisa I., Funa N., Horinouchi S. (2007). One-pot synthesis of genistein from tyrosine by coincubation of genetically engineered Escherichia coli and Saccharomyces cerevisiae cells. Appl. Microbiol. Biotechnol..

[113] Li H., Lyv Y., Zhou S., Yu S., Zhou J. (2022). Microbial cell factories for the production of flavonoids–barriers and opportunities. Bioresour. Technol..

